# Tensor Decomposition-Based Unsupervised Feature Extraction Can Identify the Universal Nature of Sequence-Nonspecific Off-Target Regulation of mRNA Mediated by MicroRNA Transfection

**DOI:** 10.3390/cells7060054

**Published:** 2018-06-04

**Authors:** Y.-H. Taguchi

**Affiliations:** Department of Physics, Chuo University, Tokyo 112-8551, Japan; tag@granular.com; Tel.: +81-3-3817-1791

**Keywords:** tensor decomposition, miRNA transfection, sequence-nonspecific off-target regulation

## Abstract

MicroRNA (miRNA) transfection is known to degrade target mRNAs and to decrease mRNA expression. In contrast to the notion that most of the gene expression alterations caused by miRNA transfection involve downregulation, they often involve both up- and downregulation; this phenomenon is thought to be, at least partially, mediated by sequence-nonspecific off-target effects. In this study, I used tensor decomposition-based unsupervised feature extraction to identify genes whose expression is likely to be altered by miRNA transfection. These gene sets turned out to largely overlap with one another regardless of the type of miRNA or cell lines used in the experiments. These gene sets also overlap with the gene set associated with altered expression induced by a Dicer knockout. This result suggests that the off-target effect is at least as important as the canonical function of miRNAs that suppress translation. The off-target effect is also suggested to consist of competition for the protein machinery between transfected miRNAs and miRNAs in the cell. Because the identified genes are enriched in various biological terms, these genes are likely to play critical roles in diverse biological processes.

## 1. Introduction

MicroRNA (miRNA) is short noncoding (functional) RNA whose primary function is mRNA degradation and disruption of translation [1]. Thus, it is generally expected that the primary effect of miRNA transfection (or overexpression) on mRNA expression is suppression. Based on this assumption, numerous miRNA transfection and/or overexpression experiments have been conducted to identify genes that are directly targeted by miRNAs [2]; during these analyses, only genes with expression levels inversely related to those of miRNA have been sought. Nevertheless, it was found that many mRNAs whose expression was likely to be altered by miRNA transfection and/or overexpression turned out to positively correlate with miRNA expression. For example, Khan et al. [3] identified multiple genes that are upregulated by miRNA transfection. They reasoned that this effect means competition with endogenous miRNAs because upregulated genes were often targeted by endogenous miRNAs. The protein machinery that binds to endogenous miRNAs was occupied by the transfected miRNAs, and as a result, the genes targeted by endogenous miRNAs were upregulated [4]. In addition, Carroll et al. [5] identified a positive correlation between mRNA expression and transfected miRNA. They theorized that the positive correlations are mediated by interactions with transcription factor E2F1.

Despite these findings, to my knowledge, sequence-nonspecific off-target regulation by miRNA transfection has not been extensively studied to date [2]. Most of the miRNA transfection and/or overexpression experiments have been aimed at identifying canonical targets of miRNAs. Most of these experiments have not been analyzed in the context of sequence-nonspecific off-target regulation by miRNA transfection. Although it is unclear why no one has tried to systematically investigate sequence-nonspecific off-target regulation mediated by miRNA transfection, one possible reason is the lack of a suitable methodology. By definition, miRNA transfection experiments cannot be composed of many samples. Typically, a pair of data points consists of miRNA-transfected cells and mock-transfected cells. Although a few more biological and/or technical replicates are possible, the number of samples available is usually less than 10. This number is often too small to detect significantly altered expression of mRNAs whose total number is up to $10^{4}$. In the case when the aim of a study is identification of canonical interactions between miRNA and mRNA, additional information that can reduce the number of mRNAs under study, e.g., bioinformatically predicted mRNAs targeted by transfected miRNAs, is available. This information can enable researchers to identify significant correlations between transfected miRNAs and mRNAs. Nonetheless, this kind of information is usually not available for the analysis of sequence-nonspecific off-target regulation by miRNA transfection.

In this study, with the aim to resolve this difficulty, I applied tensor decomposition (TD)-based unsupervised feature extraction (FE) to miRNA transfection experiments. TD-based unsupervised FE [6,7,8,9,10] is an extension of principal component analysis (PCA)-based unsupervised FE [11,12,13,14,15,16,17,18,19,20,21,22,23,24,25,26,27,28,29,30,31,32,33], which can identify critical genes even when there is only a small number of samples. TD-based unsupervised FE can also identify critical genes by means of a small number of samples available. Because of the use of this methodology, genes whose mRNA expression was likely to be altered by miRNA transfection were identified here in various combinations of cell lines and transfected miRNAs. Most of sets of genes significantly overlapped with one another regardless of transfected cell types and types of miRNA. These genes also showed altered mRNA expression under the influence of a Dicer knockout (KO). This finding suggests that the primary factor that mediated sequence-nonspecific side effects of miRNA transfection is competition for protein machinery with endogenous miRNAs, as suggested by Khan et al. [3]. In addition, these sets of genes significantly overlapped with various sets of genes whose biological functions and significance have been validated experimentally. Thus, sequence-nonspecific off-target regulation caused by miRNA transfection is expected to also play critical roles in various biological processes.

## 2. Materials and Methods

### 2.1. Mathematical Formulation of the Tensor and Tensor Decomposition

Because a tensor or tensor decomposition (TD) is not a popular mathematical concept, I briefly formulate them here. Suppose a three-mode tensor, $x_{ijk}\in R^{N\times M\times K}$, is the expression level of the *i*th mRNA when the *j*th miRNA is transfected into the *k*th sample. Samples are typically composed of biological replicates and/or treated and untreated (or mock-treated, i.e., control) samples, but situations vary. $x_{ijk}$ can also be formulated as two-mode tensor $x_{i(jk)}$, where (jk) represents a pair of a miRNA and a sample, especially when samples are not paired. $x_{ijk}$ can be decomposed to

$$x_{ijk}=\sum_{\ell_{1},\ell_{2},\ell_{3}}G\left(\ell_{1},\ell_{2},\ell_{3}\right)x_{\ell_{1}i}x_{\ell_{2}j}x_{\ell_{3}k},$$

where $G\left(\ell_{1},\ell_{2},\ell_{3}\right)\in R^{N\times M\times K}$ is a core tensor, $x_{\ell_{1}i}\in R^{N\times N},x_{\ell_{2}j}\in R^{M\times M}$ and $x_{\ell_{3}k}\in R^{K\times K}$ are singular value matrices that are orthogonal. Because this construct is obviously overcomplete, there is no unique TD. In this paper, I employed higher-order singular value decomposition [34] (HOSVD) to perform TD.

### 2.2. Using TD-Based Unsupervised FE for Identification of Genes Whose Expression Is Likely to Be Altered by MiRNA Transfection

**Figure 1:**
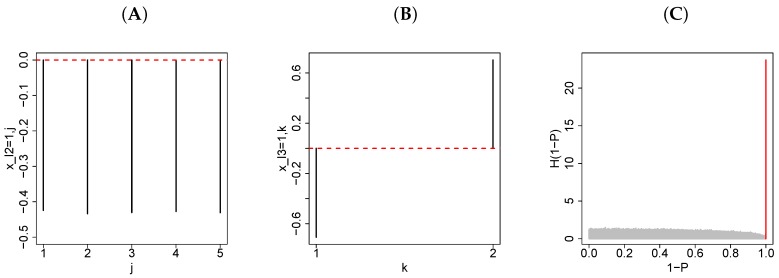
The results on the artificial data: (**A**) $x_{\ell_{2}=1,j}$ averaged across 100 independent trials. The horizontal red dashed line is $x_{\ell_{2}=1,j}=0$ (**B**) $x_{\ell_{3}=1,k}$ averaged across 100 independent trials. The horizontal red dashed line is $x_{\ell_{3}=1,k}=0$ (**C**) A histogram of 1−P computed from $x_{\ell_{1}=1,i}$. A vertical red segment represents the bin with the smallest *P*-values.

To this end, first I need to specify which sample singular value vectors, $x_{\ell_{3}k}$, have different values between treated (i.e., miRNA-transfected) samples and control (e.g., mock-transfected) samples. Suppose $x_{\ell_{3}^{\prime }k}$ turned out to represent a dissimilarity between a treated sample and control sample in some ways (see Figure 1B as an example). Next, I need to find miRNA singular value vectors, $x_{\ell_{2}j}$, that have constant values for all *j* (see Figure 1A as an example) because I would like to find genes affected constantly by miRNA transfection independently of the type of transfected miRNA, since it should represent sequence-nonspecific off-target regulation. Let us assume that $x_{\ell_{2}^{\prime }j}$ fulfilled this requirement. Then, I rank core tensors $G(\ell_{1},\ell_{2}^{\prime },\ell_{3}^{\prime })$ in the order of absolute values (largest at the top). This approach enables me to select $\ell_{1}$ such that $x_{\ell_{1}i}$ is associated with constant sequence-nonspecific off-target regulation. After identifying $\ell_{1}^{\prime }$ as those associated with larger absolute values of $G(\ell_{1}^{\prime },\ell_{2}^{\prime },\ell_{3}^{\prime })$, *i*s representing larger absolute values of $x_{\ell_{1}^{\prime }i}$ were selected. For this purpose, *P*-values, $P_{i}$s (see Figure 1C as an example), were assigned to each *i* assuming that $x_{\ell_{1}^{\prime }i}$ obeys the $\chi^{2}$ distribution

$$P_{i}=P_{\chi^{2}}\left[>\sum_{\ell_{1}^{\prime }}{\left(\frac{x_{\ell_{1}^{\prime }i}}{\sigma_{\ell_{1}^{\prime }}}\right)}^{2}\right]$$

where $P_{\chi^{2}}\left[>x\right]$ is cumulative probability that the argument is greater than *x*, assuming the $\chi^{2}$ distribution and that $\sigma_{\ell_{1}^{\prime }}$ is the standard deviation. The number of degrees of freedom of the $\chi^{2}$ distribution is equal to the number of $\ell_{1}^{\prime }$s in the summation. The above equation means that I presume that $x_{\ell_{1}^{\prime }i}$ obeys a multiple Gaussian distribution (null hypothesis). Then, $P_{i}$s were adjusted via the Benjamini–Hochberg (BH) criterion [35]. Genes associated with adjusted $P_{i}<0.01$ were finally selected (see the red bin in Figure 1C as an example).

### 2.3. Explanatory Discussion of TD-Based Unsupervised FE

Readers may wonder why a simple procedure using TD successfully identifies genes whose expression is likely to be altered by sequence-nonspecific off-target regulation mediated by various transfected miRNAs. This result can be explained as follows. Let us say most $x_{ijk}$s are a random number while a limited number of $x_{ijk}$s, e.g., $x_{i^{\prime }jk}$s are coexpressed, i.e.,

$$x_{i^{\prime }jk}=x_{jk},i^{\prime }=1,\ldots ,N^{\prime }$$

Then, the contribution of $x_{i^{\prime }jk}$ has the order of magnitude of $N^{\prime }$, while that of other $N-N^{\prime }$ (random) $x_{ijk},i\neq i^{\prime }$ has the order of magnitude of $\sqrt{N-N^{\prime }}$. Thus, even if $N^{\prime }\ll N$, if $N^{\prime }\approx \sqrt{N-N^{\prime }}$, then the contribution of $x_{i^{\prime }jk}$ outperforms that of $x_{ijk},i\neq i^{\prime }$. Thus, the contribution of $x_{i^{\prime }jk}$ should be detected as a singular value vector, $x_{\ell_{1}i}$ within which $x_{\ell_{1}i^{\prime }}$ should have a greater contribution than the others ($x_{\ell_{1}i},i\neq i^{\prime }$). Given that the contribution of $x_{\ell_{1}i},i\neq i^{\prime }$ is expected to be a Gaussian distribution, $x_{\ell_{1}i^{\prime }}$ can be detected as outliers that do not follow the Gaussian distribution. This is the possible explanation why simple TD-based unsupervised FE successfully identified genes whose expression was likely to be altered by sequence-nonspecific off-target regulation mediated by various transfected miRNAs.

### 2.4. Artificial Data

To demonstrate the usefulness of TD-based unsupervised FE, I prepared artificial dataset $x_{ijk}\in R^{N\times M\times 2}$, where *N* is the number of genes, and *M* is the number of samples (k=1 for control and k=2 for treated samples). Each treated sample is supposed to be transfected with distinct miRNA, and each control sample is supposed to be either untransfected or transfected with mock miRNA. In these artificial data, at first $x_{ijk}\in N\left(0,1\right)$. After that, they are ordered such that $x_{ijk}>x_{i^{\prime }jk}$ when $i>i^{\prime }$ with fixed *j* and *k*. This situation introduces complete correlations between distinct pairs of *j*s and *k*s (e.g., rank correlation coefficients between $x_{ijk}$ and $x_{ij^{\prime }k^{\prime }},j\neq j^{\prime },k\neq k^{\prime }$ are always equal to 1.0). Then, $x_{ijk},i>N_{0}$ were shuffled at fixed *j* and *k* to eliminate the correlation. Next, $x_{ijk}\leftarrow \left(a-1\right)x_{ijk}+a\epsilon ,\epsilon \in N\left(0,1\right),a>0$ for $i\leq N_{0}$ in order to introduce randomness into correlating rows. After that, $x_{ij2}\leftarrow -x_{ij2}$ to introduce a difference between control and treated samples. Finally, $\frac{N_{0}}{2}<i\leq N_{0}$ were shuffled at fixed *j* to generate a sample-specific difference between control and treated samples. This means that $1\leq i\leq \frac{N_{0}}{2}$ correspond to a sample-nonspecific (i.e., independent of *j*) dissimilarity between control and treated samples that represents sequence-nonspecific off-target regulation, and $\frac{N_{0}}{2}<i\leq N_{0}$ correspond to a sample-specific (i.e., dependent on *j*) dissimilarity between control and treated samples that represents sequence-specific regulation. The task at hand is to identify $1\leq i\leq \frac{N_{0}}{2}$ as precisely as possible. Specifically, $N=2000,M=5,N_{0}=50,a=0.5$.

### 2.5. Gene Expression Profiles

In this subsection, I explain 11 analyzed profiles of gene expression (Table 1) in more detail. All of them were retrieved from Gene Expression Omnibus (GEO) [36]. In some cases (Experiments 1, 2, 3, and 5), I used a two-mode tensor, $x_{i(jk)}$, which is simply denoted as $x_{ij}$, instead of three-mode tensor $x_{ijk}$, by expanding the second (*j*) and the third (*k*) modes into one column (jk) because matched data were not available. In this case, HOSVD is equivalent to simple singular value decomposition. $x_{ijk}$ and $x_{i(jk)}$ were standardized as $\sum_{i}x_{ijk}=\sum_{i}x_{i(jk)}=0$ and $\sum_{i}x_{ijk}^{2}=\sum_{i}x_{i(jk)}^{2}=N$ before TD was applied.

#### 2.5.1. No. 1: GSE26996

File GSE26996_RAW.tar was downloaded and unpacked. Six files, GSM665046_miR200a_1.txt.gz, GSM665047_miR200b_1.txt.gz, GSM665048_miR200c_1.txt.gz, GSM665049_miR200a_2.txt.gz, GSM665050_miR200b_2.txt.gz, and GSM665051_miR200c_2.txt.gz, were loaded into R using the read.csv function. Then, after the exclusion of probes with ControlType=0, gProcessedSignal and rProcessedSignal were extracted as treated and control samples, respectively. Thus, I have $x_{ij},1\leq j\leq 12,1\leq i\leq 43376$. Here, 1≤j≤6 and 7≤j≤12 are treated and control samples, respectively. For this dataset, gene expression profiles are regarded as a two-mode tensor (matrix). Applying PCA to $x_{ij}$, I found that $x_{\ell_{2}=2,j}$ are different between treated and control samples (Figure S1) independently of the type of miRNA transfected. Next, genes were selected by means of $x_{\ell_{1}=2,i}$.

#### 2.5.2. No. 2: GSE27431

File GSE27431_series_matrix.txt.gz was downloaded. It was loaded into R using the read.csv function. Each column corresponds to individual gene expression profiles named as GSEMXXXXXX, which is the GEO ID. Among those, mas5-processed samples are regarded as No. 2. GSM678153 and GSM678154 are miR-7-treated, GSM678156 and GSM678157 are miR-128-treated, whereas GSM678158, GSM678159, and GSM678160 are control samples. Then, $x_{ij},1\leq i\leq 54675,1\leq j\leq 7$ are obtained (two-mode tensor). Applying TD to $x_{ij}$, I found that $x_{\ell_{2}=2,j}$ reflects the inverse regulation between miR-128 and miR-7 while the control samples are in between (Figure S2). This finding can be interpreted as follows. Target genes of miR-128 (miR-7) are downregulated, but they are upregulated when miR-7 is transfected because of sequence-nonspecific off-target regulation caused by miRNA transfection. Accordingly, I decided to select genes using $x_{\ell_{1}=2,i}$ again.

#### 2.5.3. No. 3: GSE27431

GSE27431_series_matrix.txt.gz was again downloaded. It was loaded into R using the read.csv function. Each column corresponds to individual gene expression profiles named as GSMXXXXXX, which is a GEO ID as well. Among those, plier-processed samples are regarded as No. 3. GSM678164 and GSM678165 are miR-7-treated, GSM678167 and GSM678168 are miR-128-treated, and GSM678169, GSM678170, GSM678171, GSM678172, GSM678173, and GSM678174 are control samples. Next, $x_{ij}$, 1≤i≤54675, 1≤j≤10 are obtained (two-mode tensor). Applying PCA to $x_{ij}$, I found that $x_{\ell_{2}=2,j}$ reflects the inverse regulation between miR-128 and miR-7, while the control samples are in between (Figure S3). This result can be interpreted as in No. 2. Thus, I decided to select genes by means of $x_{\ell_{1}=2,i}$ again.

#### 2.5.4. No. 4: GSE8501

Eighteen raw data files were downloaded from GSMXXXXXX, 210896≤XXXXXX≤210913, which are GEO IDs. Columns named as INTENSITY1 and INTENSITY2 are 18 control samples and 18 samples transfected with miR-7/9/122a/128a/132/133a/142/148b/181a (two replicates each), respectively. Then, I generated tensor $x_{ijk},1\leq i\leq 23651,1\leq j\leq 18,k=1,2$, where *j*s are two replicates of each of nine miRNA-transfected samples and k=1,2 are control (mock-transfected) and transfected samples, respectively. After applying HOSVD to $x_{ijk}$, I found that $x_{\ell_{3}=2,k}$ reflects an inverse relation of expression levels between controls and treated samples, while $x_{\ell_{2}=1,j}$ reflects constant expression regardless of the type of transfected miRNA (Figure S4). Next, I decided to use $x_{\ell_{1}i}$ associated with large absolute values of $G(\ell_{1},\ell_{2}=1,\ell_{3}=2)$. Given that $G(\ell_{1}=6,\ell_{2}=1,\ell_{3}=2)$ has the largest absolute value, I decided to use $x_{\ell_{1}=6,i}$ to select mRNAs.

#### 2.5.5. No. 5: GSE41539

Four files,

GSM1018808_topo_1_empty_trimmed_RNA-Seq.txt.gz,
GSM1018809_topo_2_cel_mir_67_trimmed_RNA-Seq.txt.gz,
GSM1018810_topo_4_mir_590_3p_trimmed_RNA-Seq.txt.gz, and
GSM1018811_topo_3_mir_199a_3p_trimmed_RNA-Seq.txt.gz,

were downloaded from GSE41539. The fourth column (“Unique gene reads”) reflected gene expression. Then, I got $x_{ij},1\leq i\leq 36065,1\leq j\leq 4$ (two-mode tensor). Applying PCA to $x_{ij}$, I found that $x_{\ell_{2}=2,j}$ represents the difference between controls (mock-transfected and cel-miR-67-transfected) and miR-509/199a-3p-transfected samples (Figure S5). After that, I decided to use $x_{\ell_{1}=2,i}$ for mRNA selection.

#### 2.5.6. No. 6: GSE93290

File GSE93290_RAW.tar was downloaded and unpacked. Sixteen files, from GSM2450420 to GSM2450435, were loaded into R using the read.csv function. In each file, columns named as gProcessedSignal and rProcessedSignal served as controls and treated samples, respectively. Then, I generated three-mode tensor $x_{ijk},1\leq i\leq 62976,1\leq j\leq 16,k=1,2$, where *j*s are 16 samples and k=1,2 are control (mock-transfected) and transfected samples. After applying HOSVD to $x_{ijk}$, I found that $x_{\ell_{3}=2,k}$ reflects inversely related expression levels between controls and treated samples, whereas $x_{\ell_{2}=1,j}$ reflects constant expression regardless of the type of transfected miRNAs (Figure S6). Next, I decided to use $x_{\ell_{1}i}$ associated with large absolute values of $G(\ell_{1},\ell_{2}=1,\ell_{3}=2)$. Because $G(\ell_{1}=7,\ell_{2}=1,\ell_{3}=2)$ has the largest absolute value, I decided to apply $x_{\ell_{1}=7,i}$ to select mRNAs.

#### 2.5.7. No. 7: GSE66498

File GSE66498_RAW.tar was downloaded and unpacked. Among these data, 19 files were used, i.e., GSM1623420 to GSM1623422 (miR-205 transfected into cell lines PC3, DU145, and C4-2), GSM1623423 and GSM1623424 (miR-29a transfected into cell lines 786O and A498), GSM1623425 to GSM1623427 (miR-451/144-3p/5p transfected into the T24 cell line), GSM1623434 and GSM1623435 (24 and 48 h after miR-210 transfection into the 786O cell line), GSM1623436 to GSM1623439 (miR-145-5p/3p transfected into BOY and T24 cell lines), GSM1623440 (miR-23b transfected into 786O cells), GSM1623444 to GSM1623446 (miR-221/222/223 transfected into the PC3 cell line), and GSM1623447 (miR-223 transfected into the PC3M cell line). In each file, columns named as gProcessedSignal and rProcessedSignal served as controls and treated samples, respectively. I generated three-mode tensor $x_{ijk},1\leq i\leq 62976,1\leq j\leq 19,k=1,2$, where *j*s are the 19 samples and k=1,2 are control (mock-transfected) and transfected samples. After applying HOSVD to $x_{ijk}$, I found that $x_{\ell_{3}=2,k}$ reflects an inverse relation of expression levels between controls and treated samples, whereas $x_{\ell_{2}=1,j}$ reflects constant expression regardless of the type of transfected miRNAs (Figure S7). After that, I decided to use $x_{\ell_{1}i}$ associated with large absolute values of $G(\ell_{1},\ell_{2}=1,\ell_{3}=2)$. Because $G(\ell_{1}\in \left(2,3\right),\ell_{2}=1,\ell_{3}=2)$ have the largest and almost the same absolute values, I decided to employ $x_{\ell_{1}=2,i}$ and $x_{\ell_{1}=3,i}$ to select mRNAs.

#### 2.5.8. No. 8: GSE17759

File GSE17759_RAW.tar was downloaded and unpacked. Among these data, six replicates of miR-146a–overexpressing samples (GSM443535 to GSM443540), four replicates of miR-146b–overexpressing samples (GSM443541 to GSM443544), eight replicates of miR-146a knockout samples (GSM443557 to GSM443564), i.e., in total, 18 files were processed. In each file, columns named as gProcessedSignal and rProcessedSignal served as controls and treated samples, respectively. I generated three-mode tensor $x_{ijk},1\leq i\leq 43379,1\leq j\leq 18,k=1,2$, where *j*s are the 18 samples and k=1,2 are control (mock-transfected) and transfected samples. After applying HOSVD to $x_{ijk}$, I found that $x_{\ell_{3}=2,k}$ reflects an inverse relation of expression levels between controls and treated samples, while $x_{\ell_{2}=1,j}$ means constant expression regardless of the type of transfected miRNAs (Figure S8). Then, I decided to use $x_{\ell_{1}i}$ associated with large absolute values of $G(\ell_{1},\ell_{2}=1,\ell_{3}=2)$. Given that $G(\ell_{1}=5,\ell_{2}=1,\ell_{3}=2)$ has the largest absolute value, I decided to apply $x_{\ell_{1}=5,i}$ to select mRNAs.

#### 2.5.9. No. 9: GSE37729

Two files SE37729-GPL6098_series_matrix.txt.gz and GSE37729-GPL6104_series_matrix.txt.gz in the section “Series Matrix File(s)” were downloaded. The two files were merged such that only shared probes were included. Gene expression of HeLa cell lines is considered in Experiment No. 9. GSM926188, GSM926189, GSM926193, GSM926194, GSM926198, and GSM926201 are control samples. GSM926164 and GSM926165 are anti-miR-107-transfected samples. GSM926180, GSM926181, GSM926190, and GSM926191 are miR-107-transfected samples. GSM926162 and GSM926163 are anti-miR-181b-transfected samples. GSM926182, GSM926183, GSM926195, and GSM926196 are miR-181b-transfected samples. Then, I generated three-mode tensor $x_{ijk},1\leq i\leq 9987$, 1≤j≤6, 1≤k≤3, where k=1,2,3 correspond to control, miR-7, and miR-181b, respectively. For k=2,3, 1≤j≤2 are anti-miR-transfected samples and 3≤j≤6 are miR-transfected samples. After applying HOSVD to $x_{ijk}$, I found that $x_{\ell_{3}=2,k}$ reflects an inverse relation of expression levels between controls and treated samples, while $x_{\ell_{2}=1,j}$ indicates constant expression regardless of the type of transfected miRNA (Figure S9). Next, I decided to employ $x_{\ell_{1}i}$ associated with large absolute values of $G(\ell_{1},\ell_{2}=1,\ell_{3}=2)$. Because $G(\ell_{1}=2,\ell_{2}=1,\ell_{3}=2)$ has the largest absolute value, I decided to use $x_{\ell_{1}=2,i}$ to select mRNAs.

#### 2.5.10. No. 10: GSE37729

Two files, SE37729-GPL6098_series_matrix.txt.gz and GSE37729-GPL6104_series_matrix.txt.gz, in the section “Series Matrix File(s)” were downloaded. The two files were merged so that only shared probes are included. Gene expression of HEK 293 cell lines was considered in Experiment No. 10. GSM926206, GSM926207, GSM926211, GSM926212, GSM926216, and GSM926217 are control samples. GSM926168 and GSM926169 are anti-miR-107-transfected samples. GSM926184, GSM926185, GSM926208, and GSM926209 are miR-107-transfected samples. GSM926166 and GSM926167 are anti-miR-181b-transfected samples. GSM926186, GSM926187, GSM926213, and GSM926214 are miR-181b-transfected samples. Next, I generated three-mode tensor $x_{ijk},1\leq i\leq 9987$, 1≤j≤6, 1≤k≤3. k=1,2,3 corresponding to control, miR-7, and miR-181b, respectively. For k=2,3, 1≤j≤2 are anti-miR-transfected samples, and 3≤j≤6 are miR-transfected samples. After applying HOSVD to $x_{ijk}$, I determined that $x_{\ell_{3}=2,k}$ reflects an inverse relation of expression levels between controls and treated samples, while $x_{\ell_{2}=1,j}$ reflected constant expression regardless of the type of transfected miRNAs (Figure S10). Thus, I decided to use $x_{\ell_{1}i}$ associated with large absolute values of $G(\ell_{1},\ell_{2}=1,\ell_{3}=2)$. Because $G(\ell_{1}=2,\ell_{2}=1,\ell_{3}=2)$ has the largest absolute value, I decided to employ $x_{\ell_{1}=2,i}$ to select mRNAs.

#### 2.5.11. No. 11: GSE37729

Two files, SE37729-GPL6098_series_matrix.txt.gz and GSE37729-GPL6104_series_matrix.txt.gz, in the section “Series Matrix File(s)” were downloaded. The two files were merged such that only shared probes were included. Gene expression of SH-SY5Y cell lines was considered in Experiment No. 11. GSM926170, GSM926171, GSM926178, and GSM926179 are control samples. GSM926176 and GSM926177 are anti-miR-181b-transfected samples. GSM926174 and GSM926175 are miR-181b-transfected samples. After that, I generated three-mode tensor $x_{ijk},1\leq i\leq 9987$, 1≤j≤4, 1≤k≤2, where k=1,2 correspond to control and miR-181b, respectively. For k=2,3, 1≤j≤2 are anti-miR-transfected samples, whereas 3≤j≤6 are miR-transfected samples. After applying HOSVD to $x_{ijk}$, I found that $x_{\ell_{3}=2,k}$ reflects an inverse relation of expression levels between controls and treated samples, whereas $x_{\ell_{2}=1,j}$ reflects constant expression independently of transfected miRNAs (Figure S11). Then, I decided to use $x_{\ell_{1}i}$ associated with large absolute values of $G(\ell_{1},\ell_{2}=1,\ell_{3}=2)$. Because $G(\ell_{1}=2,\ell_{2}=1,\ell_{3}=2)$ has the largest absolute value, I decided to use $x_{\ell_{1}=2,i}$ to select mRNAs.

## 3. Results

To demonstrate the usefulness of TD-based unsupervised FE, I applied it to artificial data composed of a three-mode tensor, $x_{ijk}\in R^{N\times M\times 2}$, which is the expression level of the *i*th gene of the *j*th sample, where k=1 is control and k=2 is a treated (transfected with distinct miRNAs) sample. Among *N* genes, $N_{0}$ genes are affected by miRNA transfection while the other $N-N_{0}$ genes are not affected. Among the $N_{0}$ genes affected by miRNA transfection, $\frac{N_{0}}{2}$ genes are supposed to be regulated independently of samples (hence, a sequence-nonspecific off-target effect) while the other $\frac{N_{0}}{2}$ genes vary from sample to sample (i.e., miRNA-specific regulation). Applying TD-based unsupervised FE to the artificial dataset (averaged across 100 independent trials), I got a result (Figure 1). $x_{\ell_{2}=1,j}$ (Figure 1A) and $x_{\ell_{3}=1,k}$ (Figure 1B), which are always associated with core tensor G(1,1,1) with the largest absolute values, represent constant gene expression across *M* samples and inverted expression levels between control (k=1) and treated (k=2) samples. Accordingly, genes associated with these two are expected to represent sample- or transfected miRNA-independent (thus, sequence-nonspecific off-target) regulation. Given that $x_{\ell_{1}=1,i}$ is always associated with core tensor G(1,1,1) with the largest absolute values, *P*-values (Figure 1C) are computed using $x_{\ell_{1}=1,i}$. It is obvious that there is a sharp peak at the smallest *P*-values in the histogram of 1−P, which presumably does not correspond to the null hypothesis (that $x_{\ell_{1}=1,i}$ follows the normal distribution). To test whether genes associated with these much smaller *P*-values include genes $i\leq \frac{N_{0}}{2}$, the probabilities to be selected by TD-based unsupervised FE are averaged across $i\leq \frac{N_{0}}{2}$ and $i>\frac{N_{0}}{2}$, respectively. Then, the former is as large as 0.86, while the latter is as small as 0. (This means that genes $i>\frac{N_{0}}{2}$ have never been selected by TD-based unsupervised FE.) This observation suggests that TD-based unsupervised FE is effective at sorting out genes—that are expressed independently of samples—from genes expressed only in a limited number of samples and genes not expressed at all. To determine whether TD-based unsupervised FE can outperform the conventional supervised method, the *t* test and significance analysis of microarrays (SAM) [37] were carried out. For these two methods, *P*-values obtained were also corrected by means of the BH criterion, and genes associated with adjusted *P*-values less than 0.01 were selected. Then, the average probability for $i\leq \frac{N_{0}}{2}$ is 0.43 according to the *t* test and 0.62 according to SAM. The average probability for $i>\frac{N_{0}}{2}$ is $2\times 10^{-4}$ according to the *t* test and $3\times 10^{-5}$ according to SAM. Therefore, TD-based unsupervised FE is more effective than either the *t* test or SAM.

To identify genes whose expression alteration is likely to be mediated by sequence-nonspecific off-target regulation caused by miRNA transfection, integrated analysis of gene expression profiles after transfection of various miRNAs was performed. Simultaneous analysis of multiple experiments each of which employs single miRNA transfection will blur sequence-specific regulation of mRNAs while a sequence-nonspecific off-target effect will remain. Furthermore, to avoid biases due to research groups or individual studies, 11 experiments collected from studies involving distinct combinations of transfected miRNAs and cell lines were analyzed simultaneously (Table 1).

Genes—whose expression alteration is likely to be caused by sequence-nonspecific off-target regulation that was induced similarly by various transfected miRNAs—were selected using TD-based unsupervised FE in each of the 11 experiments. The reason why TD-based unsupervised FE was employed is as follows. First, PCA-based unsupervised FE, from which TD-based unsupervised FE was developed, is known to function even when only a small number of samples is available [16,26]. Tensor representation is also more suitable for the present experiments, where multiple genes, multiple miRNAs and controls, or transfected samples are simultaneously considered (they can be represented as a three-mode tensor; see Methods). Second, we can check whether there are miRNAs associated with a common expression pattern among all the miRNA transfection experiments by studying outcomes; we have opportunities to exclude experiments not associated with sequence-nonspecific off-target regulation caused by miRNA transfection.

These 11 analyzed experiments—in which mRNAs associated with sequence-nonspecific off-target regulation caused by miRNA transfection were successfully identified—deal with distinct cell lines into each of which distinct miRNAs were transfected. Nevertheless, gene sets identified in individual experiments not only significantly overlapped with one another but also were associated with a large enough odds ratio (from 300 to 500, Table 2), although the number of genes detected in each experiment varied from ∼100 to ∼800 (“#” in Table 2). This finding suggests that there are some sets of genes whose expression was robustly altered via sequence-nonspecific off-target regulation that was induced similarly by various transfected miRNAs.

Although this finding itself is remarkable enough to be reported, the observed coincidences may be accidental for some unknown reason and may not be associated with anything biologically valid. To validate biological significance of the identified genes, they were uploaded to Enrichr [38], which is an enrichment analysis server validating various biological terms and concepts. As a result, these genes were found to be enriched with various biological terms and concepts (see below).

First, the identified gene sets mostly included various target genes of transcription factors (TFs) (Table 3). Although the number of TFs detected varied from ∼10 to ∼100 (#2 in Table 3), the detection of multiple instances of enrichment with genes that TFs target may be evidence that these genes cooperatively function in the cell because common TFs’ target genes often have shared biological functions [39,40]. In particular, the most frequent cases of enrichment of TFs are common among the 11 experiments analyzed. These include EKLF, MYC, NELFA, and E2F1. These data can be biologically interpreted as follows.

The apparent significant alteration of expression of MYC target genes may be due to miRNAs regulating *MYC* [41,42,43]. The apparent alteration of expression of E2F1 target genes may be explained similarly because these miRNAs also target *E2F1* [41,43]. In actuality, 1551 genes targeted by only one miRNA but associated with altered gene expression caused by miRNA transfection are significantly targeted by MYC and E2F1. Enrichment with EKLF target genes among these 1551 genes not targeted by more than one miRNA—but showing altered expression caused by transfection with one of miRNAs—is a puzzle, although Yien and Beiker suggested that some miRNAs are likely also regulated by EKLF [44]. Identification of NELFA target genes is explained by the tight interaction between NELFA and c-MYC [45,46].

Additionally, I checked “TargetScan microRNA” in Enrichr to test whether enrichment with genes targeted by the transfected miRNAs would be detected. As a result, only for two of the 11 experiments (Experiments No. 2 and 3), enrichment with genes targeted by nonzero miRNAs was detected. This is possibly because $x_{\ell_{2}=2,j}$ for these two experiments also detected genes targeted by transfected miRNAs (Figures S2 and S3). In any case, the fact that most of experiments (9 out of 11) did not show enrichment with genes targeted by transfected miRNAs is consistent with the hypothesis that gene expression alteration caused by miRNA transfection is primarily due to the competition for protein machinery between endogenous miRNAs and transfected miRNAs; this pattern can remain unchanged among transfection experiments with different miRNAs.

Next, I checked KEGG pathway enrichment data. Primary enriched KEGG pathways among the identified genes are related to diseases (Table 4). Seven ((ii), (iii), (v), (vi), (vii), (viii), and (x)) out of 10 most frequently enriched KEGG pathways in the 11 experiments are directly related to various diseases. Among the remaining three ((i), (iv), and (ix)), oxidative phosphorylation is a disease-related KEGG pathway because its malfunction causes combined oxidative phosphorylation deficiency (https://www.omim.org/entry/609060). Another one, “endoplasmic reticulum”, is also a disease-related pathway because its malfunction is observed in neurological diseases [47]. Therefore, these genes may also contribute to the onset or progression of various diseases and could be therapeutic targets. As a result, sequence-nonspecific off-target regulation caused by miRNA transfection may be a therapeutic method.

Actually, gene expression alteration mediated by sequence-nonspecific off-target regulation is analogous to treatments with various candidate drugs (Table 5 and Table 6). Thus, combinatorial transfection with miRNAs may replace drug treatment in some conditions and can be used for therapeutic purposes, too. For example, LDN-192189 ((ii) in Table 5) is reported to improve neuronal conversion of human fibroblasts [48] and was proposed as a therapy for Alzheimer’s disease [49]. GSK-1059615b ((i) and (iii) in Table 5) was once considered a PI3K-kt pathway inhibitor in clinical development for the treatment of cancers [50]. WYE-125132 ((iv) in Table 5) is also known to suppress tumor growth [51] (although the name WYE-125132 does not appear in that article, compound 8a was named WYE-125132 later). Afatinib ((v) in Table 5) is a famous drug for non-small cell lung cancer [52]. PI-103 ((vi) in Table 5) is a drug for acute myeloid leukemia [53]. PD-0325901 was reported to affect heart development [54]. Chelerythrine chloride ((viii) in Table 5) has been reported to affect embryonic chick heart cells [55]. GDC-0980 ((i) in Table 6) is a known anticancer drug [56]. PLX-4720 ((ii) in Table 6) has been considered for both cancer and heart disease treatment [57]. Dinaciclib is another anticancer drug [58]. Because these are related to diseases reported in Table 4, sequence-nonspecific off-target regulation caused by miRNA transfection may be a therapeutic strategy.

Next, I studied tissue specificity of the gene expression alteration caused by sequence-nonspecific off-target regulation that was induced by miRNA transfection. Associations with GTEx tissue samples were also observed. For example, in GTEx up (Table 7), enrichment cases were observed in the brain and testes, both of which were reported to be outliers in clustering analysis [59]. Thus, it is not surprising that cases of enrichment in these two tissues were identified primarily. On the other hand, in GTEx down (Table 8), enrichment instances in the skin were mostly observed. Readers may wonder how sequence-nonspecific off-target regulation caused by miRNA transfection can contribute to the tissue specificity of gene expression profiles. Võsa et al. [60] observed that polymorphisms in miRNA response elements (MRE-SNPs) that either disrupt a miRNA-binding site or create a new miRNA-binding site can affect the allele-specific expression of target genes. Therefore, sequence-nonspecific off-target regulation caused by miRNA transfection may have the ability to contribute to tissue specificity of the gene expression profiles through distinct functionality of genetic variations in distinct tissues. Despite these coincidences, how the sequence-nonspecific off-target effect contributes to differentiation is unclear. These coincidences may simply be consequences, not causes. More studies are needed to directly implicate the sequence-nonspecific off-target effect in differentiation itself.

Genes whose expression alteration is likely to be caused by sequence-nonspecific off-target regulation that miRNA transfection induces are also enriched in pluripotency (Table 9). Because miRNAs are known to mediate reprogramming [61], sequence-nonspecific off-target regulation caused by transfection of multiple miRNAs may contribute to reprogramming too. In actuality, primary binding TFs include MYC ((i) and (iv) in Table 9) and KLF4 ((vi) and (vii) in Table 9), which are two of four Yamanaka factors that mediate pluripotency. Other TFs in Table 9 are DMAP1 (iii) and TIP60 (v). DMAP1 is a member of the TIP60-p400 complex that maintains embryonic stem cell pluripotency [62]. The remaining one, ZFX (x), has been reported to control the self-renewal of embryonic and hematopoietic stem cells [63]. In addition, two gene KO experiments have been conducted ((viii) and (ix)). One of the genes in question, *SUZ12*, encodes a polycomb group protein that mediates differentiation [64], whereas the other, *ZFP281*, is a known pluripotency suppressor [65]. There are no known widely accepted mechanisms by which miRNA transfection can induce pluripotency. Because sequence-nonspecific off-target regulation seems to alter expression of genes critical for pluripotency, it may contribute to the mechanism.

Besides, I checked whether protein–protein interactions (PPIs) are enriched in each of the 11 gene sets selected for each of the 11 experiments (Table 10). Gene sets were uploaded to the STRING server [66]. For all the gene sets, instances of PPI enrichment were highly significant. Given that proteins rarely function alone and often function in groups, this is evidence that our analysis is biologically reliable.

I demonstrated enrichment of various biological processes in gene sets. Although there were more cases of enrichment of biological terms in Enrichr, it is impossible to consider and discuss all of them. Instead, I discuss the enrichment cases identified in GeneSigDBs in Enrichr that include various biological properties (Table 11). Numerous GeneSigDBs that reflect a wide range of functional genes were enriched too. This result also suggested that the identified genes may contribute to a wide range of biological activities.

As readers can see, top four most frequently significant terms are related to either diseases or differentiation, which are often said to be biological concepts to which miRNAs contribute to. Although canonical target genes of miRNA have mainly been sought to understand biological functions of miRNAs, sequence-nonspecific off-target regulation may be important as well.

**Figure 2:**
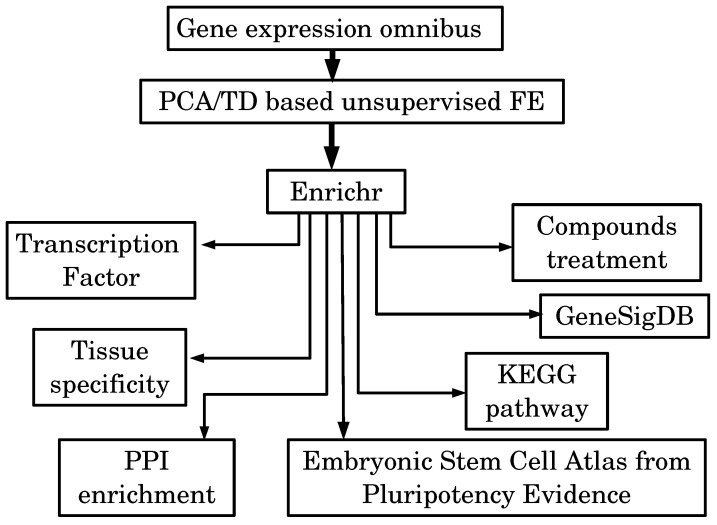
A schematic diagram that summarizes the obtained results.

Figure 2 shows the summary of results obtained in this section.

## 4. Discussion

Many biological conceptual cases of enrichment were observed in the identified sets of genes across the 11 experiments. Nonetheless, detailed mechanisms by which sequence-nonspecific off-target effects (that transfected miRNAs produce) can regulate expression of these genes are unclear. To identify such a mechanism, enrichment of genes associated with the altered expression pattern caused by a Dicer KO within these 11 gene sets was studied by means of Enrichr. Among 16 experiments included in Enrichr (“Single Gene Perturbations from GEO up” and “Single Gene Perturbations from GEO down”), most of them are associated with enrichment of the identified genes in 11 miRNA transfection experiments (Table 12). In addition, these sets of genes significantly overlap with the set of genes associated with binding to Dicer according to immunoprecipitation (IP) experiments (Table 12). There are also data supporting the hypothesis that sequence-nonspecific off-target regulation is due to the competition for protein machinery between transfected miRNAs and endogenous miRNAs in the cell.

**Figure 3:**
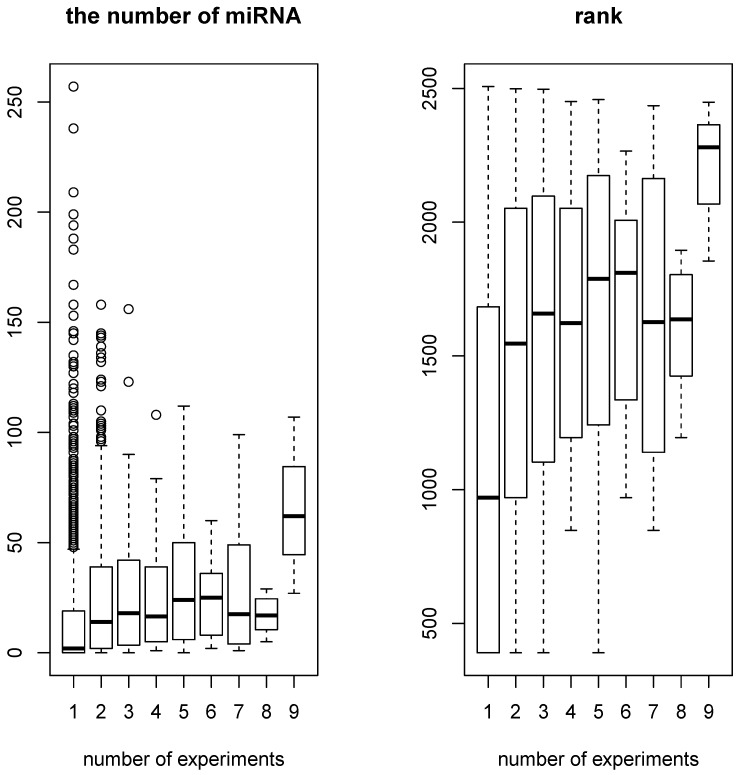
A boxplot of the number of miRNAs that target individual genes as a function of the number of experiments that select individual genes within 11 experiments (most frequently selected genes were selected in nine experiments): (**Left**) raw numbers (Pearson’s correlation coefficient = 0.13, $P=3.9\times 10^{-11}$); and (**Right**) ranks of numbers (Spearman’s correlation coefficient = 0.29, $P<2.2\times 10^{-16}$)

On the other hand, the number of miRNAs that target genes whose expression was likely to be altered by miRNA transfection significantly correlated with the number of experiments where individual genes were selected among the 11 conducted experiments (see Figure 3. Pearson’s and Spearman’s correlation coefficients are 0.13, $P=3.9\times 10^{-11}$ and 0.29, $P<2.2\times 10^{-16}$, respectively). Genes targeted by a greater number of individual miRNAs are likely to be affected by miRNA transfection, which occupies the protein machinery binding to transcripts of these genes. Therefore, the significant correlation is consistent with the hypothesis that sequence-nonspecific off-target regulation is due to competition for protein machinery between a transfected miRNA and endogenous miRNAs in cells.

Thus, primarily, gene expression alteration by sequence-nonspecific off-target regulation caused by miRNA transfection is likely due to suppression of miRNA functionality because of a reduction in the amount of available protein machinery owing to occupation by transfected miRNAs.

Despite the above arguments, it cannot be proven that competition for protein machinery is the primary cause of sequence-nonspecific off-target regulation. Upregulation of genes can also be caused by indirect effects, e.g., genes that suppress expression of other genes are repressed by transfected miRNA, although this mechanism is unlikely to cause upregulation of common genes regardless of the transfected miRNAs. Additional experimental validation will clarify which one is the correct scenario.

Furthermore, I compared the performance of TD-based unsupervised FE with the performance of major supervised methods. Previously, when PCA-based unsupervised FE, on which TD-based unsupervised FE is based, has been applied to various problems, PCA-based unsupervised FE often outperformed other (supervised) methods. For example, when PCA-based unsupervised FE was successfully used to identify genes commonly associated with aberrant promoter methylation among three autoimmune diseases [19], no supervised methods—except for PCA-based unsupervised FE—could identify common genes. On the other hand, when PCA-based unsupervised FE was applied to identify common HDACi target genes between two independent HDACis [16], two supervised methods (Limma [67] and categorical regression, i.e. analysis of variance (ANOVA)) identified the same number of common genes as did PCA-based unsupervised FE. Nevertheless, biological validation of the selected genes supported the superiority of PCA-based unsupervised FE. In contrast to the many instances of biological-term enrichment that were observed among genes selected by PCA-based unsupervised FE, such cases of enrichment among the genes selected by a supervised method were not detected.

Although these are only two examples, this kind of advantages of PCA-based unsupervised FE have often been observed. To confirm the superiority of TD-based unsupervised FE too, I consider Experiments No. 9 and No. 10 in Table 1 for the comparisons with other (supervised) methods. The reason for this choice is as follows. At first, these two were taken from the same dataset (GSE37729) and the same miRNAs (miR-107/181b) were transfected; thus, these two experiments are expected to have a greater number of common genes selected than do other pairs of experiments in Table 1. Second, the pair No. 9 and No. 10 has the highest odds ratio in Table 2, as expected. Thus, these data are suitable for the comparison with another method.

When using ANOVA and SAM [37], I found that *P*-values and the odds ratio computed by Fisher’s exact test are 0.09 and 2.9, respectively, for both methods (the number of genes selected is taken to be 103 and 104 for Experiments No. 9 and No. 10, respectively, using ranking based upon *P*-values assigned to each gene because these numbers are the same as those selected by TD-based unsupervised FE, see “#” in Table 2).

A more sophisticated and advanced supervised method may show somewhat better performance than do SAM and categorical regression. Because TD-based unsupervised FE highly outperformed these two conventional and frequently used supervised methods, it is unlikely that another supervised method can compete with TD-based unsupervised FE (advantages of PCA-based unsupervised FE over various conventional supervised methods have been reported repeatedly too [14,15,16,17,18,19,20,21,22,23,24,25,26,27,28,29,30,31,32,33]). More comprehensive performance comparisons with the *t* test are provided in the Supplementary Materials.

**Figure 4:**
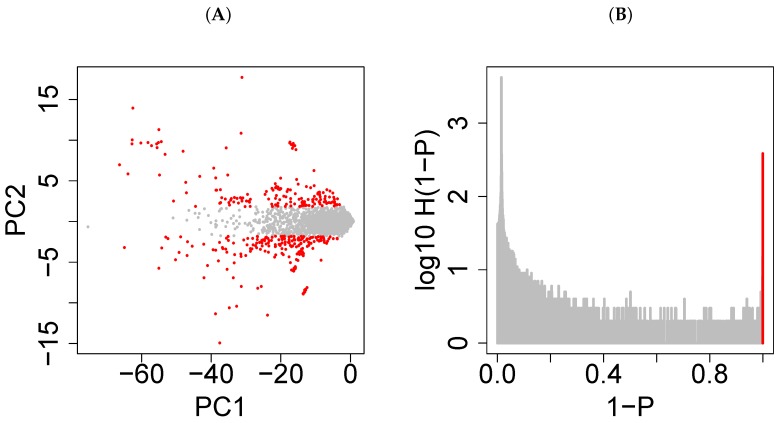
(**A**) The scatter plot of $x_{\ell_{1},i},\ell_{1}=1\left(\mathrm{horizontal}\mathrm{axis}\right),2\left(\mathrm{vertical}\mathrm{axis}\right)$ for GSE26996; 379 red dots are selected probes; and (**B**) a semilogarithmic plot of the histogram of 1−P under the null hypothesis that $x_{\ell_{1}=2,i}$ obeys a normal distribution. A sharp peak is observed in the red bin with the largest 1−P, which includes all the probes selected in (**A**).

Readers may also wonder whether the null assumption that $x_{\ell_{1},i}$ obeys a normal distribution is appropriate because $x_{\ell_{1},i}$ is not proven to follow a normal distribution. This approach is not problematic for the following reasons. First, the null hypothesis that $x_{\ell_{1},i}$ obeys a normal distribution is supposed to be rejected later. Thus, even if $x_{\ell_{1},i}$ does not follow the normal distribution, this is not a problem. Second, it is reasonable to assume that $x_{\ell_{1},i}$ follows the normal distribution under the assumption that $x_{ijk}$ is drawn from a random number (Figure 1C); this statement is suitable as the null hypothesis. Be that as it may, a question may arise whether the null hypothesis that $x_{\ell_{1},i}$ obeys the normal distribution is not suitable if this assumption is mostly violated. To evaluate how well the null hypothesis is fulfilled, I demonstrate the result for GSE26996 as a typical example. Figure 4A presents the scatter plot of $x_{\ell_{1},i},\ell_{1}=1,2$ where $x_{\ell_{1}=2,i}$ served for selection of genes as mentioned in the Section 2.5.1. Considering the fact that the total number of probes in the microarray is more than 20,000 and the number of probes selected is 379, these 379 probes are obviously outliers along the direction of $x_{\ell_{1}=2,i}$. Figure 4B depicts the histogram of 1−P under the null hypothesis that $x_{\ell_{1}=2,i}$ follows the normal distribution. Although smaller 1−P (<0.3) s deviate from constant values that are expected under the null hypothesis, a sharp peak that includes the selected 379 probes is evidently located at the largest 1−P. Consequently, it is satisfactory for identifying genes *i* associated with much larger (that is, invalidating the null hypothesis) absolute $x_{\ell_{1}=2,i}$ values.

Finally, I would like to consider possible objections to the hypothesis proposed in this study: sequence-nonspecific off-target regulation of mRNA mediated by miRNA transfection is primarily mediated by competition for the protein machinery. The first possible objection is that some miRNA can bind to a promoter region directly. This process in not mentioned in the above discussion. For example, Kim et al. [68] found that miR-320 can bind to the promoter region of *POLR3D*. Nevertheless, this kind of direct binding to DNA by transfected miRNA cannot be the interpretation of the present findings, because it is still sequence specific. Thus, direct binding to DNA cannot be an alternative interpretation of the sequence-nonspecific regulation presented in Table 2. The second objection is that miRNA can sometimes bind to mRNA with insufficient support by proteins. For example, Lima et al. [69] found that single-stranded siRNAs can bind to mRNA. Given that single-stranded siRNAs do not have to be processed by the DICER that is mentioned in Table 12, this topic apparently seems to be outside the scope of this study. Nonetheless, single-stranded siRNAs that Lima et al. identified still need the AGO2 protein. Thus, the regulation of mRNA expression by single-stranded siRNAs can still be under the control of competition for protein machinery. Therefore, it is hard to say whether the process identified by Lima et al. is independent of protein machinery competition. The third objection to the scenario proposed in this study is that miRNA can often upregulate target mRNAs [70,71]. This means that observation of upregulation caused by miRNA transfection—which was brought up as one of side proofs for protein machinery competition in the text above—does not always have to be mediated by competition for protein machinery. On the other hand, this process is also sequence specific. Thus, direct upregulation by transfected miRNA still cannot explain the sequence-nonspecific regulation of mRNAs presented in Table 2. Therefore, at the moment, protein machinery competition is the only possible explanation of the sequence-nonspecific regulation of mRNAs shown in Table 2.

## 5. Conclusions

In this study, I applied recently proposed PCA- and TD-based unsupervised FE to mRNA profiles of miRNA-transfected cell lines. mRNAs associated with significant dysregulation turned out to be independent of transfected miRNAs to some extent. This sequence-nonspecific off-target regulation is associated with various biological functions according to enrichment analysis. It is also likely to be caused by protein machinery competition between endogenous miRNAs and transfected miRNAs.

## Figures and Tables

**Table 1:** Eleven experiments conducted for this analysis. More detailed information is available in the text.

Exp.	GEO ID	Cell Lines (Cancer)	miRNA	Misc
1	GSE26996	BT549 (breast cancer)	miR-200a/b/c
2	GSE27431	HEY (ovarian cancer)	miR-7/128	mas5
3	GSE27431	HEY (ovarian cancer)	miR-7/128	plier
4	GSE8501	Hela (cervical cancer)	miR-7/9/122a/128a/132/133a/142/148b/181a
5	GSE41539	CD1 mice	cel-miR-67,hsa-miR-590-3p,hsa-miR-199a-3p
6	GSE93290	multiple	miR-10a-5p,150-3p/5p,148a-3p/5p,499a-5p,455-3p
7	GSE66498	multiple	miR-205/29a/144-3p/5p,210,23b,221/222/223
8	GSE17759	EOC 13.31 microglia cells	miR-146a/b	(KO/OE)
9	GSE37729	HeLa	miR-107/181b	(KO/OE)
10	GSE37729	HEK-293	miR-107/181b	(KO/OE)
11	GSE37729	SH-SY5Y	181b	(KO/OE)

OE: overexpression.

**Table 2:** Fisher’s exact tests for coincidence among 11 miRNA transfection experiments. Upper triangle: *P*-value; lower triangle: odds ratio.

Exp.		1	2	3	4	5	6	7	8	9	10	11
	#	232	711	747	441	123	292	246	873	113	104	120
1	232		$4.14\times 10^{19}$	$6.59\times 10^{22}$	$3.96\times 10^{41}$	$4.12\times 10^{71}$	$9.41\times 10^{70}$	$2.90\times 10^{60}$	$1.34\times 10^{17}$	$1.15\times 10^{27}$	$6.84\times 10^{26}$	$2.66\times 10^{7}$
2	711	7.68		0.00	$1.89\times 10^{18}$	$4.93\times 10^{27}$	$5.59\times 10^{20}$	$2.69\times 10^{32}$	$4.62\times 10^{13}$	$9.23\times 10^{16}$	$8.66\times 10^{12}$	$1.37\times 10^{3}$
3	747	8.30	345.52		$3.63\times 10^{20}$	$7.96\times 10^{21}$	$5.70\times 10^{12}$	$1.82\times 10^{27}$	$9.52\times 10^{12}$	$1.18\times 10^{14}$	$1.01\times 10^{12}$	$3.90\times 10^{6}$
4	441	18.23	5.19	5.34		$6.14\times 10^{41}$	$1.01\times 10^{34}$	$1.44\times 10^{69}$	$4.61\times 10^{11}$	$2.16\times 10^{30}$	$4.09\times 10^{28}$	$1.35\times 10^{10}$
5	123	53.86	9.04	7.27	17.48		$2.9\times 10^{179}$	$1.27\times 10^{63}$	$6.24\times 10^{15}$	$3.16\times 10^{25}$	$2.37\times 10^{17}$	$4.69\times 10^{9}$
6	292	61.50	8.15	5.52	17.71	204.39		$3.53\times 10^{53}$	$2.57\times 10^{15}$	$6.65\times 10^{22}$	$1.65\times 10^{12}$	$5.60\times 10^{5}$
7	246	20.27	5.35	4.67	12.39	20.11	22.03		$6.91\times 10^{42}$	$1.77\times 10^{36}$	$4.50\times 10^{31}$	$2.78\times 10^{14}$
8	873	18.61	7.22	6.51	8.29	15.61	18.53	20.73		$1.81\times 10^{7}$	$1.37\times 10^{6}$	$2.76\times 10^{2}$
9	113	39.34	9.87	8.77	25.98	32.44	34.90	21.94	16.02		$3.7\times 10^{125}$	$9.27\times 10^{18}$
10	104	40.29	8.22	8.27	26.64	23.34	20.86	21.56	15.18	517.87		$6.82\times 10^{16}$
11	120	10.15	3.19	4.43	9.19	11.55	8.11	8.28	4.92	19.57	18.70	

#: the number of genes selected for each of 11 experiments via TD- or PCA-based unsupervised FE.

**Table 3:** In each of 11 experiments, 20 top-ranked significant TFs whose sets of target genes significantly overlap with the set of genes selected for each experiment were identified. Then, EKLF, MYC, NELFA, and E2F1 turned out to be among the 20 top-ranked significant TFs for all 11 experiments.

			EKLF		MYC		NELFA		E2F1	
Exp.	#1	#2	OL	adj. *P*-Value	OL	adj. *P*-Value	OL	adj. *P*-Value	OL	adj. *P*-Value
1	232	30	40/1239	$2.16\times 10^{7}$	53/1458	$6.22\times 10^{12}$	59/2000	$8.94\times 10^{10}$	61/1529	$1.30\times 10^{15}$
2	711	77	94/1239	$1.51\times 10^{10}$	106/1458	$1.01\times 10^{10}$	134/2000	$3.26\times 10^{11}$	100/1529	$6.14\times 10^{8}$
3	747	97	100/1239	$2.28\times 10^{11}$	98/1458	$2.96\times 10^{7}$	152/2000	$1.93\times 10^{15}$	108/1529	$3.89\times 10^{9}$
4	441	43	83/1239	$4.77\times 10^{18}$	99/1458	$2.08\times 10^{22}$	105/2000	$1.06\times 10^{15}$	85/1529	$9.29\times 10^{14}$
5	123	45	26/1239	$2.16\times 10^{6}$	25/1458	$9.12\times 10^{5}$	31/2000	$4.26\times 10^{5}$	28/1529	$7.41\times 10^{6}$
6	292	19	51/1239	$2.38\times 10^{9}$	65/1458	$5.54\times 10^{14}$	63/2000	$2.72\times 10^{7}$	69/1529	$8.31\times 10^{15}$
7	246	11	37/1239	$5.11\times 10^{5}$	48/1458	$5.97\times 10^{8}$	46/2000	$1.45\times 10^{3}$	64/1529	$7.02\times 10^{16}$
8	873	55	188/1239	$8.33\times 10^{52}$	189/1458	$8.58\times 10^{42}$	222/2000	$3.52\times 10^{39}$	157/1529	$8.24\times 10^{23}$
9	113	36	24/1239	$4.47\times 10^{6}$	30/1458	$2.86\times 10^{8}$	32/2000	$2.33\times 10^{6}$	40/1529	$6.84\times 10^{15}$
10	104	22	27/1239	$1.16\times 10^{8}$	25/1458	$4.83\times 10^{6}$	36/2000	$1.07\times 10^{9}$	35/1529	$3.63\times 10^{12}$
11	120	22	21/1239	$8.02\times 10^{4}$	27/1458	$2.25\times 10^{5}$	29/2000	$4.68\times 10^{4}$	25/1529	$3.57\times 10^{4}$

#1: the number of genes selected for each of 11 experiments via TD- or PCA-based unsupervised FE; #2: the number of TFs whose sets of target genes significantly (adjusted *P*-values <0.01) overlap with the set of genes selected for each experiment; OL: overlaps, (the number of genes coinciding with the genes selected for each experiment)/(genes listed in Enrichr as TF target genes).

**Table 4:** In each of 11 experiments, 20 top-ranked significant KEGG pathways whose associated genes significantly match some genes selected for each experiment were identified. Thus, the following KEGG pathways are most frequently ranked within the top 20.

Exp.	#	(i)	(ii)	(iii)	(iv)	(v)	(vi)	(vii)	(viii)	(ix)	(x)
1	(232)	31/137	7/168	10/142	6/133	9/55	9/193			7/169	8/203
	[10]	$3.69\times 10^{29}$	$3.18\times 10^{2}$	$1.66\times 10^{4}$	$3.45\times 10^{2}$	$1.02\times 10^{6}$	$6.85\times 10^{3}$			$3.18\times 10^{2}$	$3.01\times 10^{2}$
2	(711)	36/137	18/168	14/142	12/133	13/55				16/169	18/203
	[12]	$3.43\times 10^{19}$	$1.48\times 10^{3}$	$1.05\times 10^{2}$	$3.20\times 10^{2}$	$5.92\times 10^{6}$				$8.12\times 10^{3}$	$8.12\times 10^{3}$
3	(747)	23/137	15/168			14/55				18/169	19/203
	[15]	$3.58\times 10^{7}$	$1.94\times 10^{2}$			$1.20\times 10^{6}$				$2.02\times 10^{3}$	$4.78\times 10^{3}$
4	(441)	50/137	15/168	19/142	18/133	6/55	19/193	7/78	12/151	9/169	
	[10]	$2.92\times 10^{45}$	$1.91\times 10^{4}$	$3.97\times 10^{8}$	$6.42\times 10^{8}$	$2.49\times 10^{2}$	$3.40\times 10^{6}$	$2.74\times 10^{2}$	$4.44\times 10^{3}$	$1.29\times 10^{1}$	
5	(123)	9/137						8/78		6/169	8/203
	[23]	$2.97\times 10^{6}$						$6.08\times 10^{7}$		$4.29\times 10^{3}$	$3.03\times 10^{4}$
6	(292)	45/137	20/168	19/142	18/133	4/55	19/193	11/78	12/151		
	[14]	$1.35\times 10^{46}$	$3.32\times 10^{11}$	$2.27\times 10^{11}$	$4.00\times 10^{11}$	$7.95\times 10^{2}$	$2.24\times 10^{9}$	$4.90\times 10^{7}$	$4.87\times 10^{5}$		
7	(246)	40/137	9/168	10/142	9/133		11/193	4/78	7/151		6/203
	[6]	$5.61\times 10^{42}$	$6.60\times 10^{3}$	$5.80\times 10^{4}$	$1.32\times 10^{3}$		$1.16\times 10^{3}$	$2.57\times 10^{1}$	$6.31\times 10^{2}$		$4.52\times 10^{1}$
8	(873)	75/137	30/168	32/142	32/133		36/193	14/78	24/151	25/169	
	[24]	$5.59\times 10^{63}$	$2.09\times 10^{9}$	$9.32\times 10^{13}$	$1.89\times 10^{13}$		$7.51\times 10^{12}$	$1.39\times 10^{4}$	$1.62\times 10^{6}$	$3.11\times 10^{6}$	
9	(113)	18/137	11/168	12/142	10/133	6/55	12/193	4/78	11/151		
	[20]	$8.24\times 10^{18}$	$7.10\times 10^{8}$	$1.66\times 10^{9}$	$8.42\times 10^{8}$	$8.85\times 10^{6}$	$2.96\times 10^{8}$	$6.64\times 10^{3}$	$2.96\times 10^{8}$		
10	(104)	11/137	8/168	9/142	8/133	5/55	10/193		8/151		
	[20]	$1.98\times 10^{8}$	$6.68\times 10^{5}$	$3.23\times 10^{6}$	$1.71\times 10^{5}$	$1.56\times 10^{4}$	$3.23\times 10^{6}$		$3.60\times 10^{5}$		
11	(120)	6/137		4/142		5/55					5/203
	[3]	$9.04\times 10^{3}$		$8.49\times 10^{2}$		$2.98\times 10^{3}$					$6.83\times 10^{2}$

(i) Ribosome: hsa03010; (ii) Alzheimer’s disease: hsa05010; (iii) Parkinson’s disease: hsa05012; (iv) Oxidative phosphorylation: hsa00190; (v) Pathogenic *Escherichia coli* infection:hsa05130; (vi) Huntington’s disease: hsa05016; (vii) Cardiac muscle contraction: hsa04260; (viii) Nonalcoholic fatty liver disease (NAFLD): hsa04932; (ix) Protein processing in endoplasmic reticulum: hsa04141; and (x) Proteoglycans in cancer: hsa05205. (numbers): gene; [numbers]: KEGG pathways. Upper rows in each exp: (the number of genes coinciding with the genes selected for each experiment)/(genes listed in Enrichr in each category). Lower rows in each exp: adjusted *P*-values provided by Enrichr.

**Table 5:** In each of 11 experiments, 20 top-ranked significant treatments with compounds whose downregulated genes significantly coincide with some genes selected for each experiment were identified. Thus, treatments with the following compounds are most frequently ranked within top 20.

Exp.	#		(i)	(ii)	(iii)	(iv)	(v)	(vi)	(vii)	(viii)
1	(232)	OL	10/85				16/154			
	[129]	adj *P*-value	$6.14\times 10^{5}$				$6.59\times 10^{7}$			
2	(711)	OL								
	[329]	adj *P*-value								
3	(747)	OL							
	[417]	adj *P*-value								
4	(441)	OL	16/85		14/105	15/109		16/144		
	[67]	adj *P*-value	$8.56\times 10^{7}$		$1.15\times 10^{4}$	$5.10\times 10^{5}$		$1.56\times 10^{4}$		
5	(123)	OL		17/141			12/154			
	[219]	adj *P*-value		$2.87\times 10^{13}$			$2.26\times 10^{7}$			
6	(292)	OL			13/105					19/137
	[132]	adj *P*-value			$9.69\times 10^{6}$					$3.04\times 10^{9}$
7	(246)	OL	9/85				12/154			
	[61]	adj *P*-value	$1.41\times 10^{3}$				$8.88\times 10^{4}$			
8	(873)	OL	30/85	46/141					35/162	
	[255]	adj *P*-value	$1.19\times 10^{15}$	$1.62\times 10^{23}$					$5.21\times 10^{12}$	
9	(119)	OL	9/85	11/141		8/109		8/144	8/162	12/137
	[74]	$6.25\times 10^{6}$	$3.48\times 10^{6}$		$3.50\times 10^{4}$		$1.45\times 10^{3}$	$2.33\times 10^{3}$	$2.76\times 10^{7}$	
10	(104)	OL	9/85		9/105	9/109		10/144		12/137
	[155]	adj *P*-value	$1.87\times 10^{6}$		$4.96\times 10^{6}$	$6.22\times 10^{6}$		$4.96\times 10^{6}$		$7.01\times 10^{8}$
11	(120)	OL		10/141					9/162	
	[127]	adj *P*-value		$4.74\times 10^{5}$					$5.26\times 10^{4}$	

(i) LJP005_BT20_24H-GSK-1059615-3.33; (ii) LJP005_HS578T_24H-LDN-193189-10; (iii) LJP005_MCF10A_24H-GSK-1059615-10; (iv) LJP006_BT20_24H-WYE-125132-10; (v) LJP006_HS578T_24H-afatinib-10; (vi) LJP006_MCF10A_24H-PI-103-10; (vii) LJP007_HT29_24H-PD-0325901-0.12; and (viii) LJP009_HEPG2_24H-chelerythrine_chloride-10. #: (numbers): genes; [numbers]: compounds. Upper rows in each exp: OL, overlaps, (the number of genes coinciding with the genes selected for each experiment)/(genes listed in Enrichr in each category). Lower rows in each exp: adjusted *P*-values provided by Enrichr.

**Table 6:** In each of the 11 experiments, 20 top-ranked significant treatments with compounds whose sets of upregulated genes significantly overlap with the set of genes selected for each experiment were identified. Therefore, treatment with the following compounds is most frequently ranked within the top 20.

Exp.	#	(i)	(ii)	(iii)
1	(232)			16/166
	[191]			$2.16\times 10^{7}$
2	(711)	23/125	30/163	
	[450]	$1.58\times 10^{7}$	$1.11\times 10^{9}$	
3	(747)		34/163	
	[559]		$6.14\times 10^{12}$	
4	(441)	15/125		
	[85]	$2.12\times 10^{4}$		
5	(123)			
	[116]			
6	(292)		18/163	
	[190]		$2.46\times 10^{7}$	
7	(246)			
	[145]			
8	(873)	22/125		31/166
	[69]	$6.56\times 10^{5}$		$1.80\times 10^{7}$
9	(119)			8/166
	[0]			$1.95\times 10^{2}$
10	(104)			
	[0]			
11	(120)			
	[33]			

(i) LJP005_A375_24H-GDC-0980-0.37; (ii) LJP005_HEPG2_24H-PLX-4720-10; and (iii) LJP007_MCF7_24H-dinaciclib-0.12 #: (numbers): genes; [numbers]: compounds. Upper rows in each exp: OL, overlaps (the number of genes coinciding with the genes selected for each experiment)/(genes listed in Enrichr in each category). Lower rows in each exp: adjusted *P*-values provided by Enrichr.

**Table 7:** In each of the 11 experiments, 20 top-ranked significant tissues whose set of upregulated genes significantly overlapped with the set of genes selected for each experiment were identified.

Exp.	#	(i)	(ii)	(iii)	(iv)	(v)	(vi)	(vii)	(viii)	(ix)	(x)
1	(232)	68/1509	51/1066	65/1384	74/1773	77/1764		69/1770		53/1174	68/1710
	[216]	$1.11\times 10^{20}$	$3.65\times 10^{16}$	$1.28\times 10^{20}$	$8.31\times 10^{21}$	$8.66\times 10^{23}$		$8.65\times 10^{18}$		$8.12\times 10^{16}$	$7.14\times 10^{18}$
2	(711)						78/625				
	[470]						$3.13\times 10^{20}$				
3	(747)						72/625				
	[441]						$1.93\times 10^{15}$				
4	(441)	80/1509	61/1066	82/1384		87/1764	54/625		54/525		
	[151]	$3.20\times 10^{11}$	$1.01\times 10^{9}$	$7.82\times 10^{14}$		$1.08\times 10^{5}$	$6.23\times 10^{15}$		$6.83\times 10^{18}$		
5	(123)	31/1509	29/1066	32/1384	33/1773			34/1770		26/1174	33/1710
	[150]	$2.48\times 10^{7}$	$9.29\times 10^{9}$	$1.75\times 10^{8}$	$5.68\times 10^{7}$			$1.99\times 10^{7}$		$9.17\times 10^{7}$	$2.90\times 10^{7}$
6	(292)	80/1509	66/1066	70/1384	80/1773	85/1764	46/625	78/1770	50/525	63/1174	76/1710
	[196]	$7.02\times 10^{22}$	$3.72\times 10^{21}$	$4.91\times 10^{18}$	$4.91\times 10^{18}$	$4.97\times 10^{21}$	$2.53\times 10^{17}$	$4.73\times 10^{17}$	$4.99\times 10^{23}$	$2.53\times 10^{17}$	$7.45\times 10^{17}$
7	(246)	64/1509	53/1066	57/1384	68/1773	68/1764	34/625	63/1770	35/525	52/1174	63/1710
	[135]	$7.77\times 10^{16}$	$1.13\times 10^{15}$	$1.02\times 10^{13}$	$3.42\times 10^{15}$	$3.28\times 10^{15}$	$5.23\times 10^{11}$	$1.16\times 10^{12}$	$1.03\times 10^{13}$	$1.10\times 10^{13}$	$2.75\times 10^{13}$
8	(873)	164/1509	131/1066	156/1384	155/1773	168/1764		161/1770		128/1174	157/1710
	[178]	$1.61\times 10^{25}$	$8.18\times 10^{25}$	$1.61\times 10^{25}$	$2.25\times 10^{15}$	$4.48\times 10^{20}$		$2.02\times 10^{17}$		$1.31\times 10^{19}$	$2.38\times 10^{17}$
9	(119)	39/1509	28/1066	40/1384	38/1773	37/1764	24/625		25/525		
	[225]	$1.13\times 10^{13}$	$8.60\times 10^{10}$	$2.22\times 10^{15}$	$5.07\times 10^{11}$	$1.92\times 10^{10}$	$3.31\times 10^{11}$		$1.13\times 10^{13}$		
10	(104)	29/1509	22/1066	30/1384	31/1773	29/1764		28/1770	15/525	23/1174	27/1710
	[156]	$2.83\times 10^{7}$	$5.82\times 10^{6}$	$1.93\times 10^{8}$	$4.52\times 10^{7}$	$4.31\times 10^{6}$		$1.19\times 10^{5}$	$1.35\times 10^{5}$	$5.82\times 10^{6}$	$1.51\times 10^{5}$
11	(120)						13/625		12/525		
	[5]						$1.54\times 10^{2}$		$1.38\times 10^{2}$		

(i) GTEX-QDT8-0011-R10A-SM-32PKG_brain_female_30-39_years; (ii) GTEX-QMR6-1426-SM-32PLA_brain_male_50-59_years; (iii) GTEX-TSE9-3026-SM-3DB76_brain_female_60-69_years; (iv) GTEX-PVOW-0011-R3A-SM-32PKX_brain_male_40-49_years; (v) GTEX-PVOW-2526-SM-2XCF7_brain_male_40-49_years; (vi) GTEX-XAJ8-1326-SM-47JYT_testis_male_40-49_years; (vii) GTEX-N7MS-0011-R3a-SM-33HC6_brain_male_60-69_years; (viii) GTEX-OHPM-2126-SM-3LK75_testis_male_50-59_years; (ix) GTEX-PVOW-0011-R5A-SM-32PL7_brain_male_40-49_years; and (x) GTEX-PVOW-2626-SM-32PL8_brain_male_40-49_years. (numbers): gene; [numbers]: tissues. *P*-values are the ones adjusted by Enrichr.

**Table 8:** In each of the 11 experiments, 20 top-ranked significant tissues whose set of downregulated genes significantly overlapped with the set of genes selected for each experiment were identified.

Exp.	#	(i)	(ii)	(iii)	(iv)	(v)	(vi)
1	(232)	57/1709	54/1488	53/1027	53/1121	55/1599	52/1103
	[201]	$4.88\times 10^{11}$	$1.40\times 10^{11}$	$4.65\times 10^{17}$	$1.13\times 10^{15}$	$4.65\times 10^{11}$	$1.96\times 10^{15}$
2	(711)	166/1709			133/1121	175/1599	
	[414]	$4.72\times 10^{32}$			$1.27\times 10^{33}$	$2.48\times 10^{40}$	
3	(747)	174/1709				165/1599	
	[365]	$1.44\times 10^{33}$				$2.20\times 10^{32}$	
4	(441)	98/1709	89/1488	73/1027		98/1599	70/1103
	[219]	$2.41\times 10^{16}$	$8.07\times 10^{16}$	$2.17\times 10^{16}$		$5.24\times 10^{18}$	$1.38\times 10^{13}$
5	(123)			35/1027	35/1121		37/1103
	[486]			$9.87\times 10^{15}$	$1.04\times 10^{13}$		$2.03\times 10^{15}$
6	(292)		69/1488	60/1027	53/1121		61/1103
	[171]		$2.30\times 10^{15}$	$3.96\times 10^{17}$	$5.96\times 10^{12}$		$9.15\times 10^{17}$
7	(246)		55/1488	57/1027	49/1121	53/1599	51/1103
	[113]		$2.46\times 10^{11}$	$7.80\times 10^{19}$	$1.81\times 10^{12}$	$3.39\times 10^{9}$	$1.38\times 10^{13}$
8	(873)	188/1709		137/1027	136/1121	182/1599	138/1103
	[185]	$1.15\times 10^{30}$		$8.37\times 10^{30}$	$1.34\times 10^{25}$	$3.79\times 10^{31}$	$3.16\times 10^{27}$
9	(119)	31/1709	36/1488				
	[224]	$4.11\times 10^{7}$	$6.46\times 10^{11}$				
10	(104)	31/1709	33/1488				
	[156]	$3.24\times 10^{8}$	$1.64\times 10^{10}$				
11	(120)						
	[20]						

(i) GTEX-Q2AH-0008-SM-48U2J_skin_male_40-49_years; (ii) GTEX-O5YT-0126-SM-48TBW_skin_male_20-29_years; (iii) GTEX-P4PQ-0008-SM-48TDX_skin_male_60-69_years; (iv) GTEX-R55D-0008-SM-48FEV_skin_male_50-59_years; (v) GTEX-R55E-0008-SM-48FCG_skin_male_20-29_years; and (vi) GTEX-RU72-0008-SM-46MV8_skin_female_50-59_years. (numbers): gene; [numbers]: tissues. Upper rows in each exp: OL, overlaps (the number of genes coinciding with the genes selected for each experiment)/(genes listed in Enrichr in each category). Lower rows in each exp: adjusted *P*-values provided by Enrichr.

**Table 9:** In each of the 11 experiments, 20 top-ranked significant terms in the Embryonic Stem Cell Atlas from Pluripotency Evidence (ESCAPE) whose set of associated genes significantly overlapped with the set of genes selected for each experiment were identified.

Exp.	#	(i)	(ii)	(iii)	(iv)	(v)	(vi)	(vii)	(viii)	(ix)	(x)
1	(232)	53/1458	90/2469	55/1789	56/1200	24/705	38/1700	40/1502	15/315	17/186	66/3249
	[15]	$3.85\times 10^{12}$	$1.63\times 10^{22}$	$7.36\times 10^{10}$	$1.35\times 10^{17}$	$7.38\times 10^{5}$	$1.40\times 10^{3}$	$2.40\times 10^{5}$	$1.13\times 10^{4}$	$2.84\times 10^{9}$	$6.10\times 10^{5}$
2	(711)	106/1458	184/2469	96/1789	90/1200				33/315		182/3249
	[22]	$1.29\times 10^{10}$	$2.83\times 10^{21}$	$5.46\times 10^{4}$	$1.09\times 10^{9}$				$1.43\times 10^{6}$		$4.08\times 10^{9}$
3	(747)	98/1458	199/2469	106/1789	93/1200	55/705			32/315	19/186	184/3249
	[28]	$4.59\times 10^{7}$	$7.29\times 10^{25}$	$2.60\times 10^{5}$	$1.88\times 10^{9}$	$8.48\times 10^{6}$			$1.09\times 10^{5}$	$1.17\times 10^{3}$	$9.00\times 10^{8}$
4	(441)	99/1458	153/2469	109/1789	95/1200	51/705	64/1700	60/1502		23/186	145/3249
	[18]	$1.37\times 10^{22}$	$1.91\times 10^{32}$	$1.74\times 10^{21}$	$2.31\times 10^{26}$	$3.51\times 10^{12}$	$3.44\times 10^{4}$	$1.21\times 10^{4}$		$8.71\times 10^{10}$	$1.39\times 10^{16}$
5	(123)	25/1458	46/2469	26/1789					19/315		
	[32]	$3.65\times 10^{5}$	$9.91\times 10^{11}$	$3.38\times 10^{4}$					$1.66\times 10^{11}$		
6	(292)	65/1458	108/2469	54/1789	59/1200	24/705	47/1700	37/1502	13/315	19/186	68/3249
	[8]	$2.14\times 10^{14}$	$3.68\times 10^{25}$	$1.03\times 10^{5}$	$1.26\times 10^{14}$	$3.96\times 10^{3}$	$6.28\times 10^{4}$	$2.48\times 10^{2}$	$1.89\times 10^{2}$	$2.10\times 10^{9}$	$2.34\times 10^{2}$
7	(246)	48/1458	78/2469	43/1789	49/1200	17/705	31/1700	33/1502	13/315	15/186	
	[6]	$2.16\times 10^{8}$	$1.87\times 10^{13}$	$6.95\times 10^{4}$	$8.53\times 10^{12}$	$1.48\times 10^{1}$	$2.48\times 10^{1}$	$2.70\times 10^{2}$	$5.72\times 10^{3}$	$6.01\times 10^{7}$	
8	(873)	189/1458	303/2469	184/1789	159/1200	89/705	130/1700	116/1502	38/315	38/186	247/3249
	[28]	$5.44\times 10^{42}$	$6.58\times 10^{67}$	$1.81\times 10^{27}$	$6.07\times 10^{36}$	$4.59\times 10^{18}$	$4.78\times 10^{9}$	$2.74\times 10^{8}$	$3.67\times 10^{7}$	$4.46\times 10^{14}$	$1.89\times 10^{18}$
9	(119)	30/1458	54/2469	37/1789	28/1200	16/705	23/1700	28/1502		9/186	32/3249
	[14]	$1.76\times 10^{8}$	$4.54\times 10^{18}$	$1.22\times 10^{10}$	$6.09\times 10^{9}$	$6.01\times 10^{5}$	$1.46\times 10^{3}$	$5.60\times 10^{7}$		$3.47\times 10^{5}$	$1.15\times 10^{2}$
10	(104)	25/1458	56/2469	33/1789	21/1200	13/705	23/1700	19/1502		10/186	
	[11]	$3.77\times 10^{6}$	$3.80\times 10^{22}$	$4.45\times 10^{9}$	$2.91\times 10^{5}$	$1.53\times 10^{3}$	$4.39\times 10^{4}$	$4.56\times 10^{3}$		$2.87\times 10^{6}$	
11	(120)	27/1458	44/2469		17/1200	13/705	23/1700	25/1502	7/315		
	[14]	$1.28\times 10^{5}$	$1.60\times 10^{9}$		$1.20\times 10^{2}$	$5.75\times 10^{3}$	$4.08\times 10^{3}$	$1.97\times 10^{4}$	$3.00\times 10^{2}$		

(i) CHiP_MYC-19079543; (ii) mESC_H3K36me3_18692474; (iii) CHiP_DMAP1-20946988; (iv) CHiP_MYC-18555785; (v) CHiP_TIP60-20946988; (vi) CHiP_KLF4-18358816; (vii) CHiP_KLF4-19030024; (viii) SUZ12-17339329_UP; (ix) ZFP281-21915945_DOWN; and (x) CHiP_ZFX-18555785. (numbers): gene; [numbers]: TF binding, histone modification and a gene KO or overexpression. Upper rows in each exp: OL, overlaps (the number of genes coinciding with the genes selected for each experiment)/(genes listed in Enrichr in each category). Lower rows in each exp: adjusted *P*-values provided by Enrichr.

**Table 10:** PPI enrichment by the STRING server. Column “genes” shows numbers of genes recognized by the STRING server.

Exp.	Genes	Edges		*P*-Values
		**Observed**	**Expected**	
1	195	1638	591	0
2	623	4271	2577	0
3	658	4506	2920	0
4	392	3418	1273	0
5	118	539	197	0
6	276	2048	569	0
7	182	1167	303	0
8	640	10176	4342	0
9	112	464	165	0
10	103	323	127	0
11	118	143	104	$1.58\times 10^{4}$

**Table 11:** In each of the 11 experiments, 20 top-ranked significant terms in GeneSigDB whose set of associated genes significantly overlapped with the set of genes selected for each experiment were identified.

			(i)		(ii)		(iii)		(iv)	
Exp.	#1	#2	OL	adj. *P*-Value	OL	adj. *P*-Value	OL	adj. *P*-Value	OL	adj. *P*-Value
1	232	154	97/1548	$7.60\times 10^{44}$	36/663	$1.68\times 10^{12}$	77/2585	$1.72\times 10^{13}$	36/238	$6.63\times 10^{27}$
2	711	194	152/1548	$4.03\times 10^{29}$	72/663	$3.28\times 10^{15}$	222/2585	$8.96\times 10^{36}$	44/238	$3.05\times 10^{17}$
3	747	285	149/1548	$4.32\times 10^{25}$	87/663	$1.58\times 10^{22}$	222/2585	$4.01\times 10^{32}$	30/238	$2.05\times 10^{7}$
4	441	106	146/1548	$7.43\times 10^{52}$	62/663	$6.57\times 10^{20}$	145/2585	$1.51\times 10^{25}$	46/238	$2.15\times 10^{27}$
5	123	183	44/1548	$4.46\times 10^{16}$	13/663	$1.84\times 10^{3}$	37/2585	$1.34\times 10^{5}$	24/238	$1.54\times 10^{19}$
6	292	106	103/1548	$1.21\times 10^{38}$	36/663	$1.79\times 10^{9}$	94/2585	$1.51\times 10^{15}$	39/238	$5.50\times 10^{27}$
7	246	95	81/1548	$5.23\times 10^{28}$	30/663	$1.34\times 10^{7}$	66/2585	$3.88\times 10^{7}$	30/238	$2.86\times 10^{19}$
8	873	269	256/1548	$1.82\times 10^{81}$	100/663	$8.26\times 10^{26}$	229/2585	$4.03\times 10^{25}$	86/238	$7.47\times 10^{53}$
9	119	93	60/1548	$6.98\times 10^{34}$	30/663	$9.55\times 10^{17}$	48/2585	$9.73\times 10^{13}$	22/238	$4.26\times 10^{18}$
10	104	77	52/1548	$1.67\times 10^{27}$	25/663	$1.59\times 10^{12}$	45/2585	$4.60\times 10^{12}$	15/238	$2.49\times 10^{10}$
11	120	68	36/1548	$6.11\times 10^{10}$	17/663	$4.25\times 10^{5}$	40/2585	$1.07\times 10^{6}$	13/238	$6.73\times 10^{7}$

(i) A multiclass predictor based on a probabilistic model, i.e., application to gene expression profiling-based diagnosis of thyroid tumors; (ii) A redox signature score identifies diffuse large B-cell lymphoma patients with a poor prognosis; (iii) A comparison of the gene expression profile of undifferentiated human embryonic stem cell lines and differentiating embryoid bodies; and (iv) A gene expression profile of rat left ventricles reveals persisting changes after a chronic mild-exercise protocol: implications for cardioprotection. #1: genes; #2: terms. OL: overlaps (the number of genes coinciding with the genes selected for each experiment)/(genes listed in Enrichr in each category).

**Table 12:** GEO DICER KO: The number of experiments among the 16 experiments included in Enrichr whose set of listed genes significantly overlapped with the set of genes identified in each of the 11 experiments. IP: Fisher’s exact test for the overlap between the set of genes that bind to Dicer in immunoprecipitation (IP) experiments and the set of genes selected in each of the 11 experiments.

**Experiments**		**1**	**2**	**3**	**4**	**5**	**6**
GEO DICER KO	up	12/16	12/16	12/16	12/16	14/16	11/16
	down	13/16	12/16	12/16	13/16	14/16	10/16
IP	*P*-value	$2.49\times 10^{23}$	$7.22\times 10^{22}$	$1.31\times 10^{17}$	$5.55\times 10^{29}$	$5.21\times 10^{35}$	$1.78\times 10^{20}$
	odds	47.4	20.6	15.9	38.7	64.2	41.2
**Experiments**			**7**	**8**	**9**	**10**	**11**
GEO DICER KO	up		12/16	14/16	12/16	13/16	12/16
	down		12/16	12/16	14/16	14/16	10/16
IP	*P*-value		$4.72\times 10^{32}$	$4.29\times 10^{16}$	$2.19\times 10^{11}$	$3.96\times 10^{10}$	$4.64\times 10^{8}$
	odds		37.0	41.4	42.6	39.6	27.3

## Supplementary Materials

The following files are available online at http://www.mdpi.com/2073-4409/7/6/54/s1, Supplementary Document: the *t* test applied to a real dataset; Supplementary Data: a full list of the identified genes; Supplementary Figures: Figures S1–S11.
