# Supplementary material for: Tensor Decomposition-Based Unsupervised Feature Extraction Can Identify the Universal Nature of Sequence-Nonspecific Off-Target Regulation of mRNA Mediated by MicroRNA Transfection
Source: Cells. 2018 Jun 4;7(6):54. doi: 10.3390/cells7060054 (PMC6025034; doi:10.3390/cells7060054)
Supplement: Supplementary file 1 [file cells-07-00054-s001.zip › Supp.pdf]

Supporting information : Performances of t test applied to real data

In order to demonstrate how t test is inferior to TD and PCA based unsupervised FE, t test was applied to 11 real data set to which TD or PCA based unsupervised FE was applied in the main text. In this analysis, samples are divided into two classes: samples to which no miRNAs (or mock miRNA) were transfected and samples to which miRNA transfected. Hereafter, we call these as control and treated samples, respectively. Two sided t test was applied to each of 11 data set with using t.test function in R. Then, obtained P-values were adjusted with using p.adjust function in R with assigning "BH" option in order to specify BH criterion. Then, probes associated with adjusted P-values less than 0.01 were selected (Table S13).

Table S13: The number of genes selected by t test. The numbering of experiments are the same as those in main paper. Two numbers besides colon are the number of control and treated samples, respectively.

Experiments	1	2	3	4	5	6	7	8	9	10	11
Samples	6:6	3:4	6:4	18:18	2:2	16:16	19:19	18:18	6:12	6:12	4:4
Selected genes	11060	0	0	0	0	35	280	55	5949	5730	0

The result is a little bit disappointing. For five out of 11 experiments, t test cannot identify any differently expressed genes. On the other hand, the numbers of selected genes vary from 35 to 11060. These number are unlikely biologically trustable. This possibly shows the failure of methodology.

In order to further demonstrate the inferiority of t test to TD or PCA based unsupervised FE, we try to reproduce a table that corresponds to Table S2. Since the number of genes selected by t test are often 0 (Table S13), the selected genes by t test are taken to be as many as those in Table S2 (i.e., # in Table S2) for the comparison (Table S14).

Table S14: The coincidence of genes selected by t test between 11 experiments. Notations are the same as those in Table S2.

	1	2	3	4	5	6	7	8	9	10	11
1		4.96e-04	8.49e-01	2.59e-01	6.35e-01	1.00e+00	5.40e-01	1.00e+00	4.08e-01	6.45e-01	6.68e-01
2	2.56		6.40e-69	1.38e-02	1.25e-01	1.55e-01	9.36e-03	1.00e+00	1.00e+00	3.76e-01	1.00e+00
3	0.80	10.49		8.65e-01	5.28e-01	3.76e-01	2.47e-01	7.79e-01	7.75e-01	5.30e-01	1.00e+00
4	1.55	1.90	0.89		6.58e-01	1.00e+00	4.31e-01	1.26e-01	2.71e-01	2.56e-01	1.00e+00
5	0.00	0.00	0.36	1.39		1.13e-22	1.00e+00	3.86e-01	1.00e+00	1.00e+00	1.00e+00
6	0.77	1.83	0.32	0.72	27.05		3.71e-01	1.00e+00	1.00e+00	1.00e+00	1.00e+00
7	1.16	0.48	0.71	1.22	0.67	0.31		4.47e-01	1.83e-01	7.60e-02	2.04e-01
8	0.64	1.00	1.17	2.15	2.09	0.00	0.46		1.59e-01	4.54e-01	1.27e-03
9	0.00	0.81	0.60	0.00	0.00	0.00	0.25	2.91		1.18e-03	4.07e-01
10	0.00	0.32	0.35	1.75	0.00	0.00	0.00	1.68	5.56		6.37e-01
11	1.31	0.78	0.88	0.97	0.00	0.00	1.69	6.87	0.00	0.00	

It is obvious that the selected genes by t test are less coincident with each other than the selected genes by PCA or TD based unsupervised FE (Table S2) since odds ratios are smaller and P-values are larger. Thus, also from the point of coincidence between 11 experiments, t test is inferior to TD or PCA based unsupervised FE.

One may wonder why TD and PCA based unsupervised FE result in so distinct outcomes from t test. In order to see this point, valcano plots (Fig. S16) are drawn for all genes in

11 experiments (grey dots). Black dots correspond to genes selected by TD or PCA based unsupervised FE. It is obvious that the genes selected by TD or PCA based unsupervised FE are not always associated with either larger FC or smaller P-values. Thus it is important to see what genes are selected by TD or PCA based unsupervised FE. In order to see this, gene expression profiles were overdrawn after genes selected by PCA or TD based unsupervised FE in each experiment are clustered into six clusters (Fig. S17. The most frequently selected genes are shown in black). The clustering is necessary since profile is not always shared by all selected genes (e.g., up- vs downregulated between control and treated samples). At a glance, profiles do not obey simple "stepwise" profiles that have constant values within control and treated samples while constant values differ from control and treated samples. The profiles that the genes selected by PCA or TD based unsupervised FE exhibit are obviously too complicated to be selected by simply applying t test which prefers "stepwise" shape between control and treated samples. This is the reason why genes selected by TD or PCA based unsupervised FE are not always associated with either larger FC or smaller P-values.

One may still wonder that these complicated profiles that genes selected by PCA or TD based unsupervised FE exhibit might mean the failure of TD or PCA based unsupervised FE. In contrast to the first impression, P-values computed when t test was applied to not individual genes but all genes in each cluster as a group are often very small (Fig. S17). This suggests that TD or PCA based unsupervised FE could identify differently expressed genes between control and treated samples, although selection criterion is distinct from t test that prefers stepwise shape.

One may also wonder why TD or PCA based unsupervised FE does not select genes associated with "stepwise" profile that t test prefers. The reason why the outcome of TD or PCA based unsupervised FE differs from the outcome that t test gives is because t test cannot take into account the amount of gene expression while PCA or TD based unsupervised FE can. Even if expression of a specific genes are doubled over all samples, neither P-value computed by t test nor FC can change. Nevertheless, since PCA or TD based unsupervised FE attribute P-values to gene using PC scores attributed to each gene, doubled gene expression results in smaller P-values. Thus, when TD or PCA based unsupervised FE is employed, genes associated with larger expression are more likely selected (because they have more chances to have smaller P-values under the null hypothesis of Gaussian distribution). This is a primary difference between TD or PCA based unsupervised FE from t test. Although one may be afraid that the emphasis of the amount of gene expression itself might bias the selection of genes, it results in more biologically reasonable results; genes are more coincidently selected between experiments while many biological terms are enriched in the selected genes.

As a conclusion, TD or PCA based unsupervised FE can select genes that differ from genes selected by t test. This difference is because TD or PCA based unsupervised FE emphasize the amount of gene expression itself which is ignored by FC or t test. And the emphasis of the amount of expression result in more biologically reasonable results.

Experiment No. 1

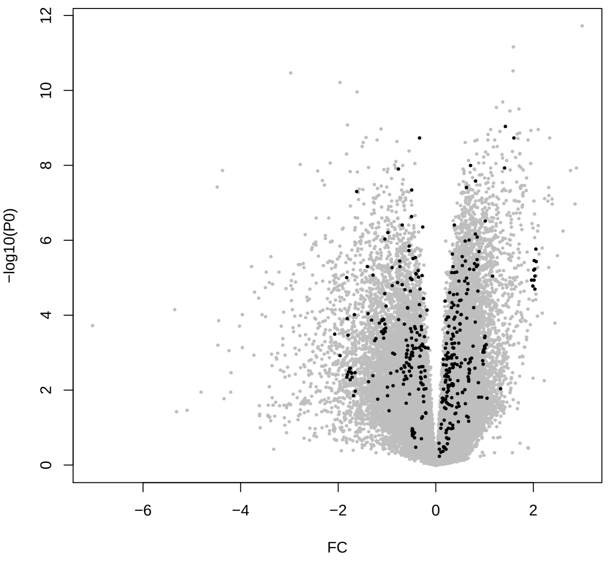

Experiment No. 2

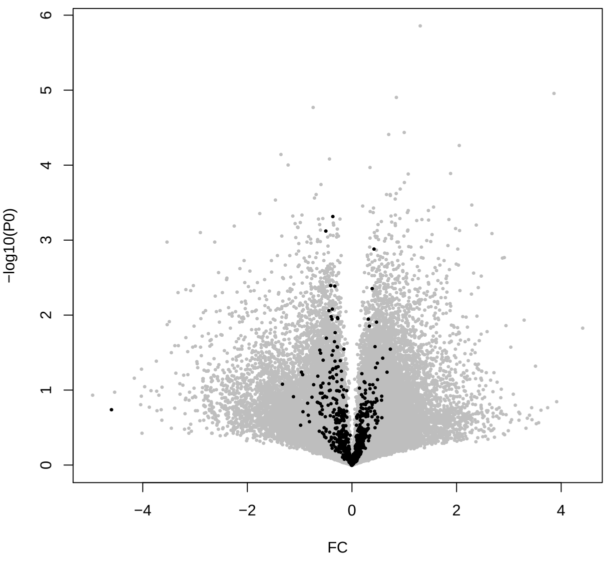

Experiment No. 3

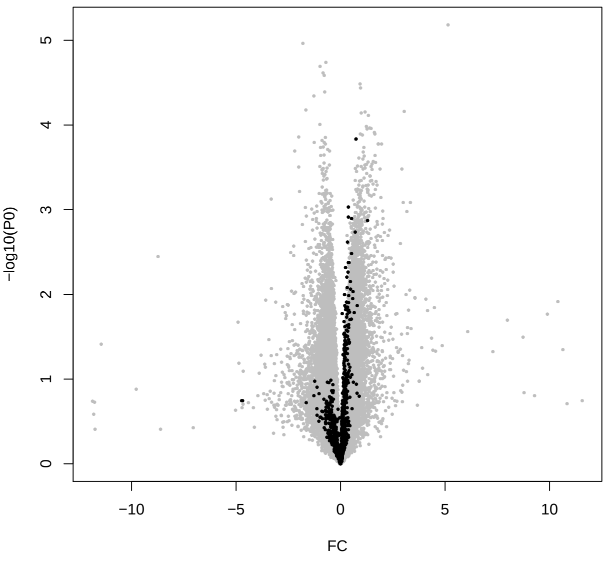

Experiment No. 4

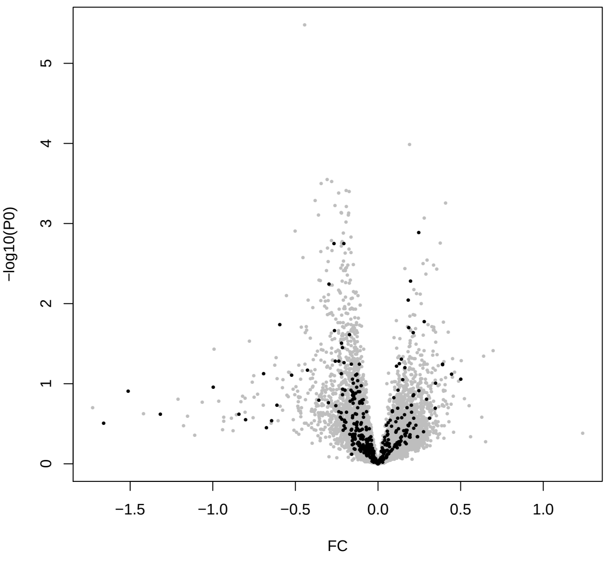

Experiment No. 5

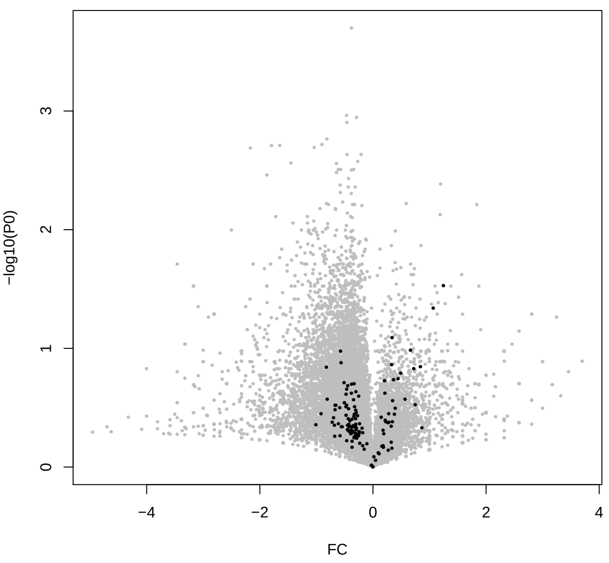

Experiment No. 6

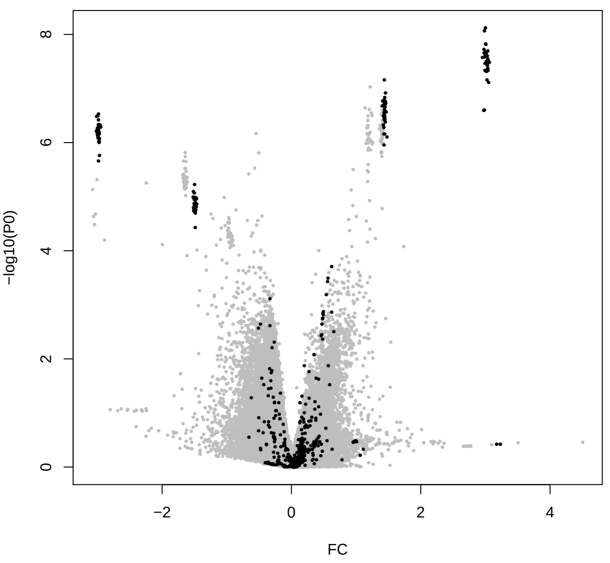

Experiment No. 7

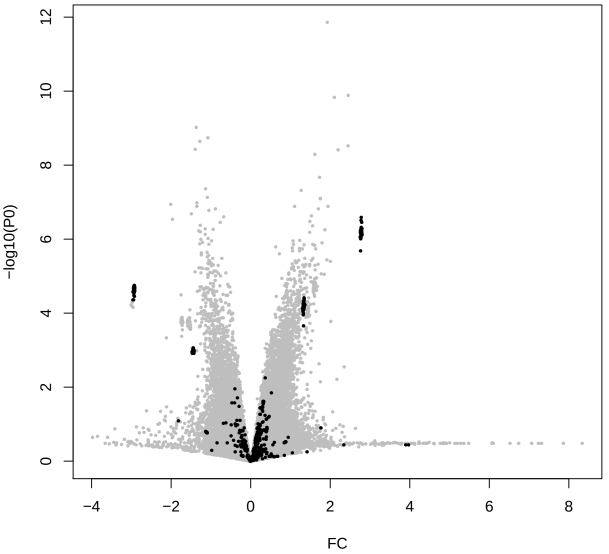

Experiment No. 8

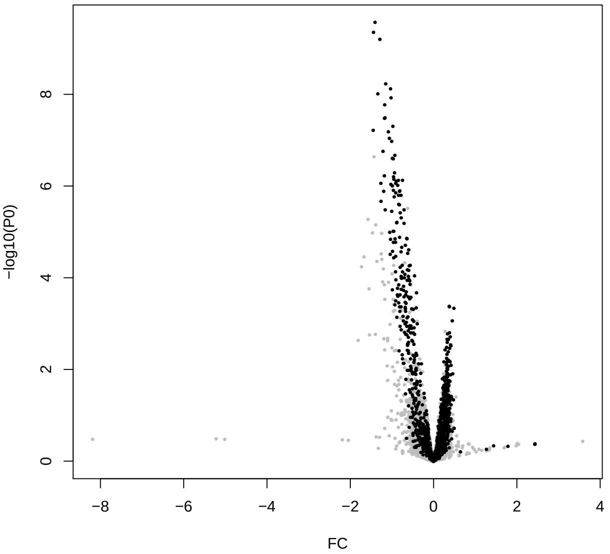

Experiment No. 9

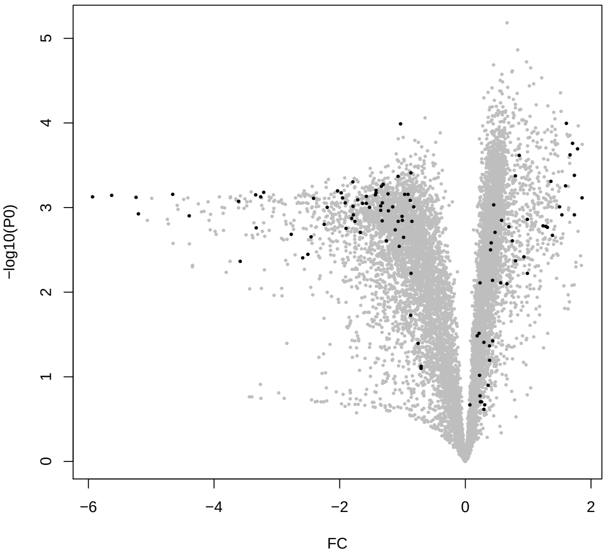

Experiment No. 10

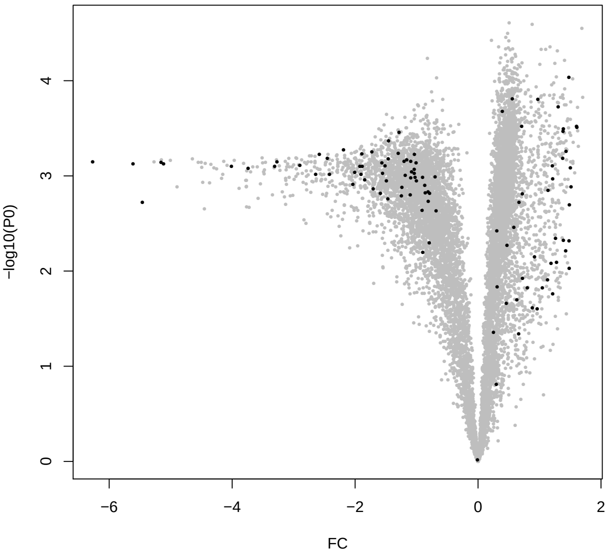

Experiment No. 11

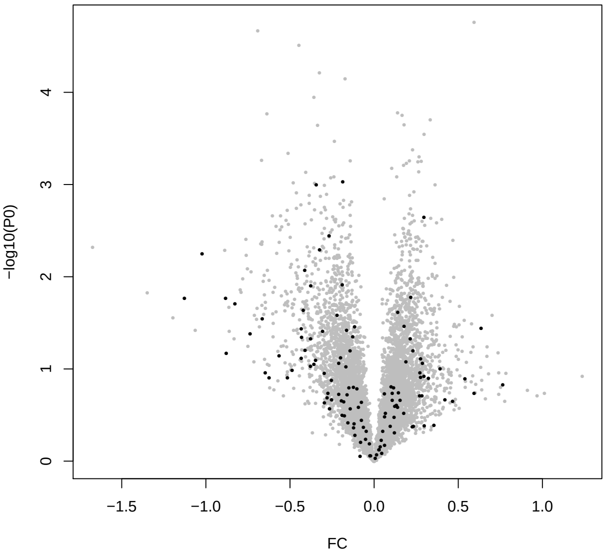

Figure S16: Valcano plots for all genes (grey dots) in each experiment. Black dots are genes selected by PCA or TD based unsupervised FE in each experiment. Vertically, $-\log_{10} P$ was plotted where P is P-value that t test attributed to each gene. FC (horizontal axis) is logarithmic fold change, defined as $\log_2 \left(\frac{\langle x_{ij} \rangle_{j \in \text{control}}}{\langle x_{ij} \rangle_{j \in \text{treated}}} \right)$. $\langle x_{ij} \rangle_{j \in S}$ represents expression of ith genes averaged over jth samples that belongs to set S. Larger FC corresponds to downregulation in treated samples than control samples and is coincident with canonical function of transfected miRNAs, i.e., suppression of target mRNAs.

Experiment No. 1

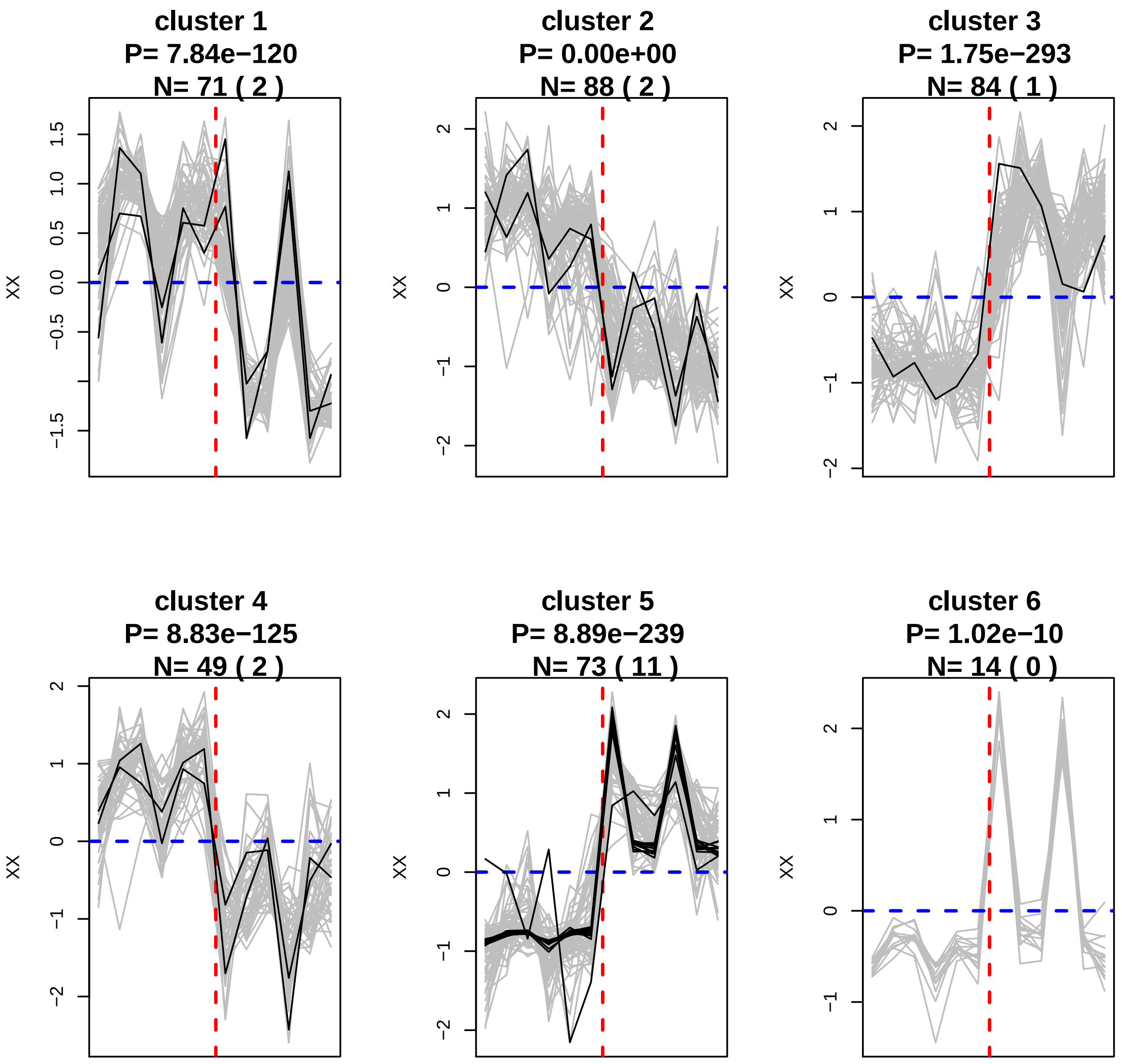

Experiment No. 2

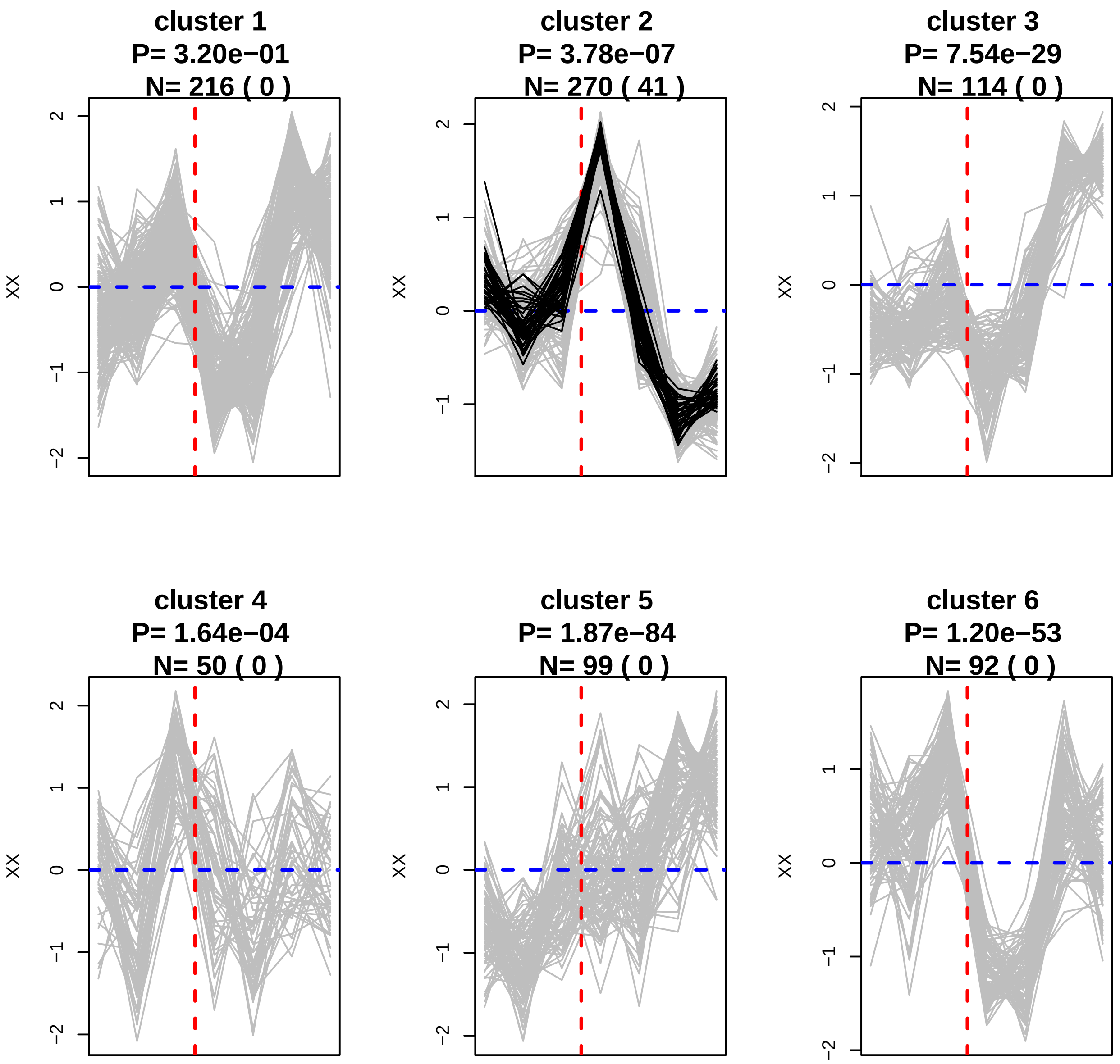

Experiment No. 3

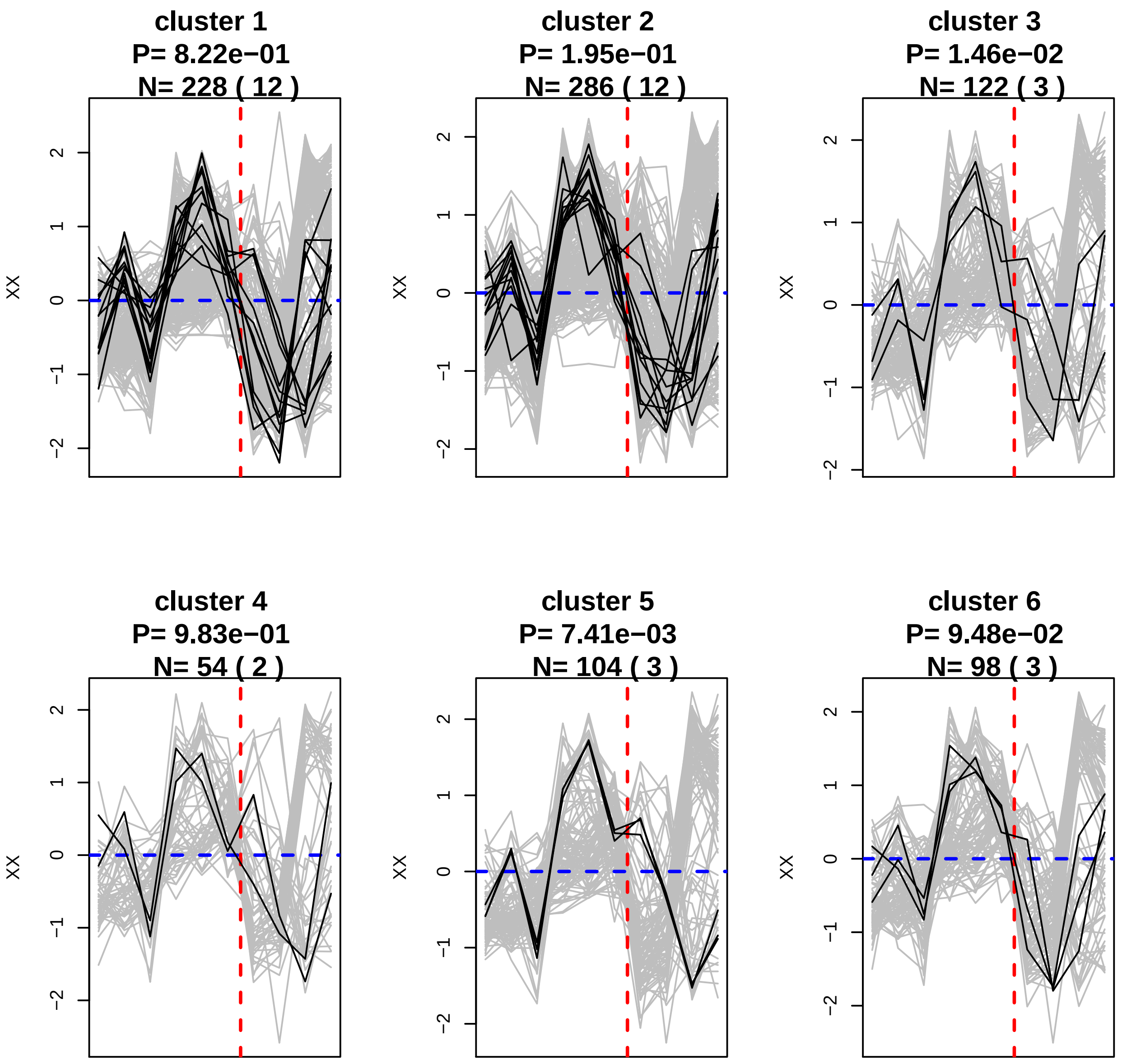

Experiment No. 4

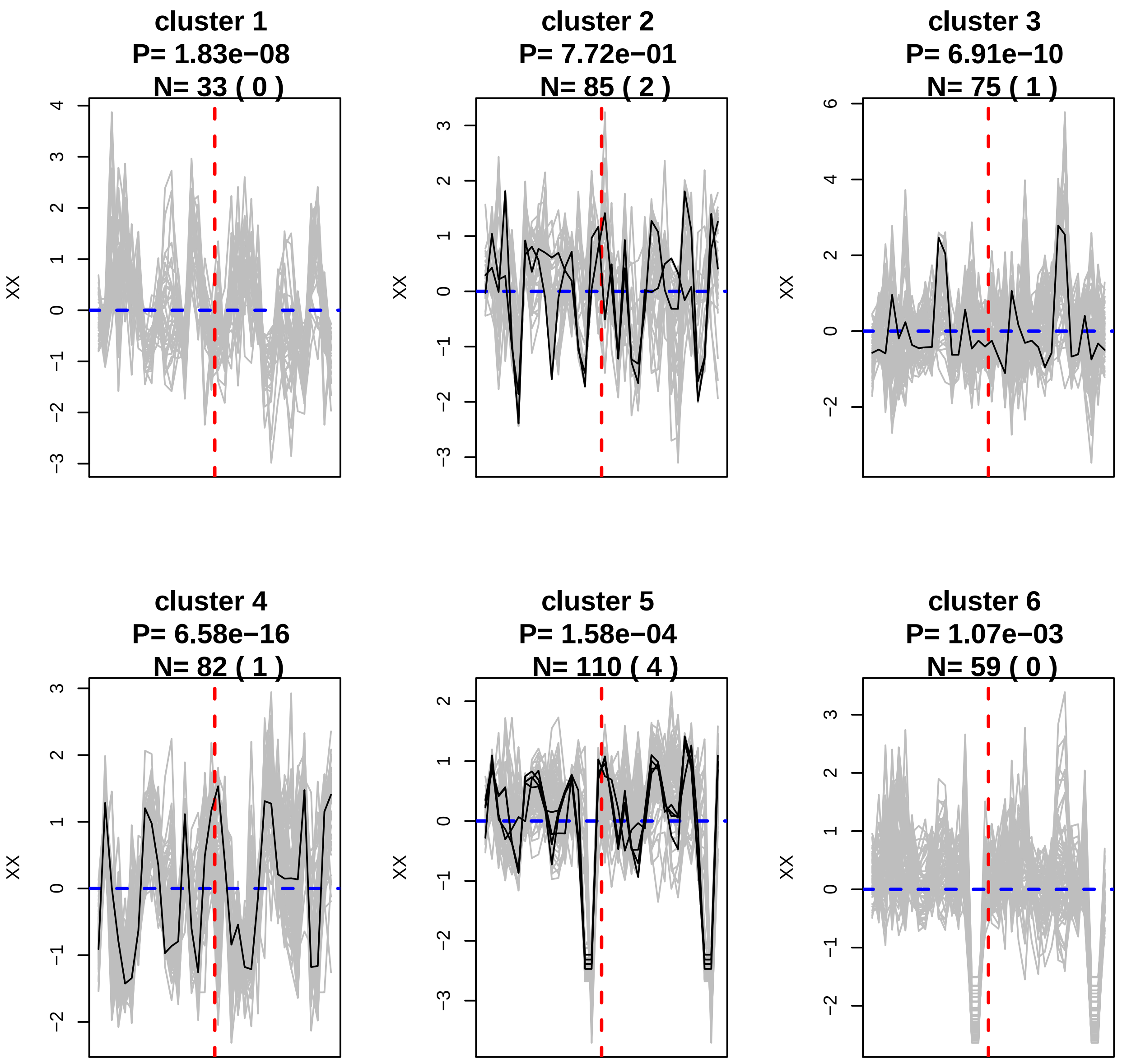

Experiment No. 5

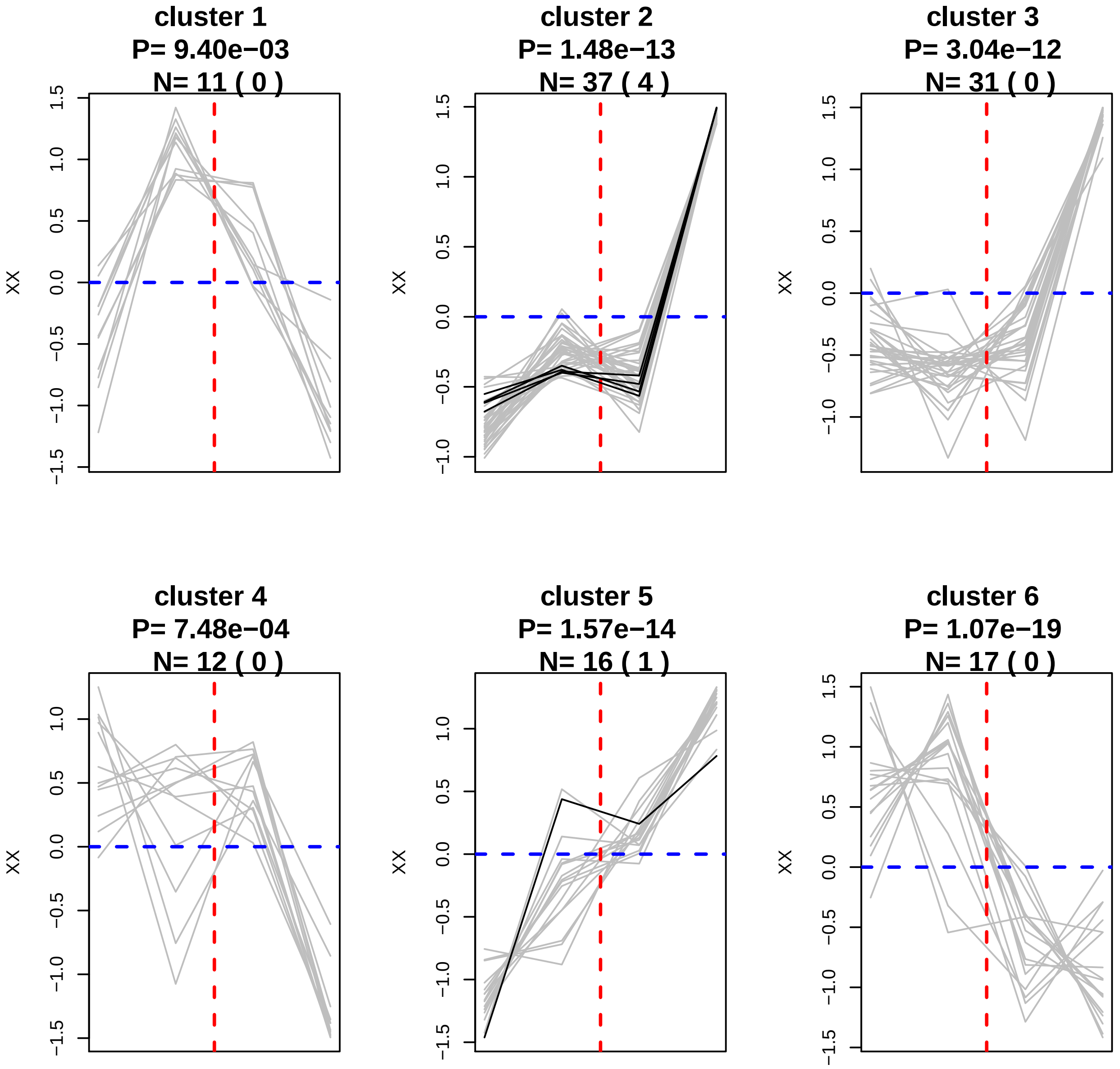

Experiment No. 6

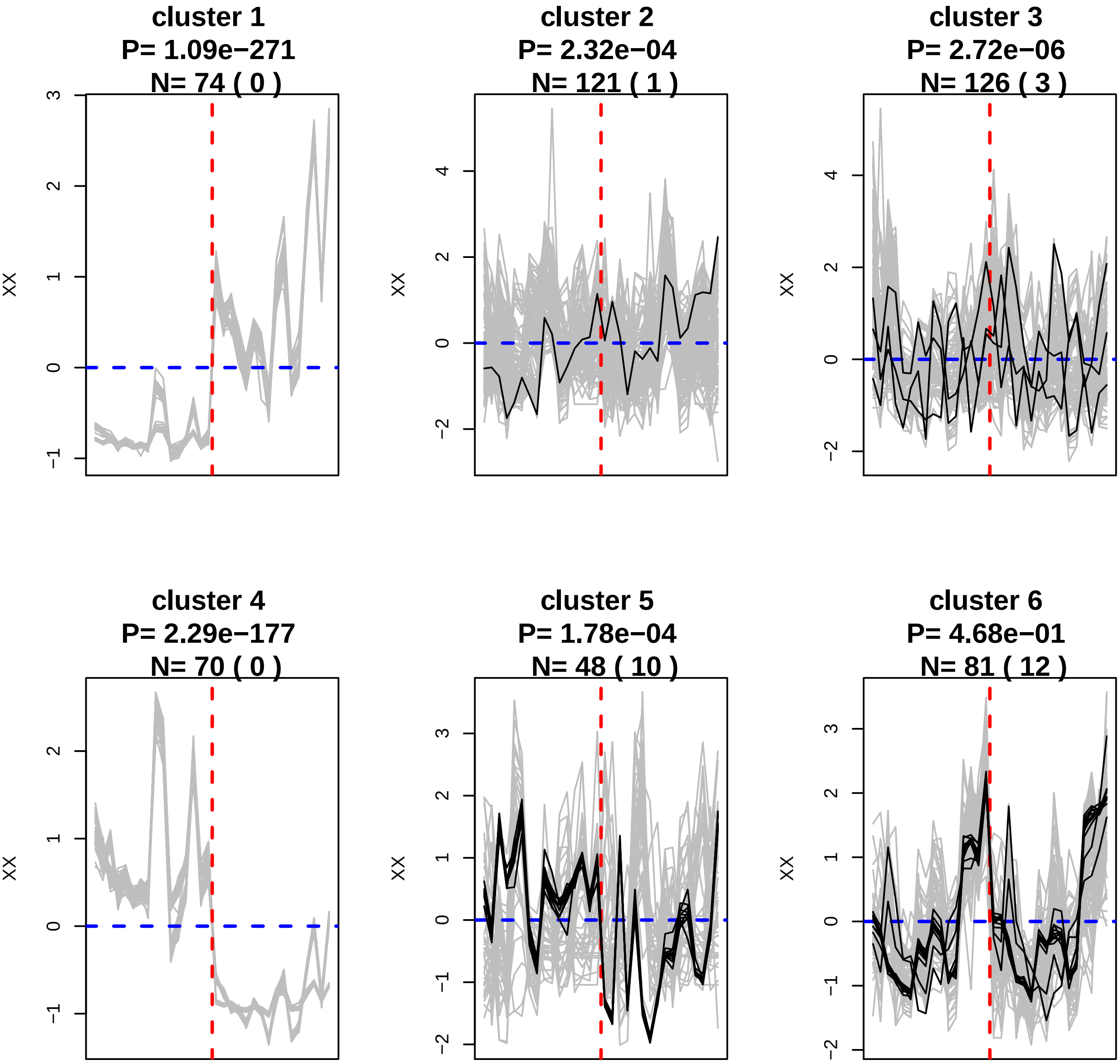

Experiment No. 7

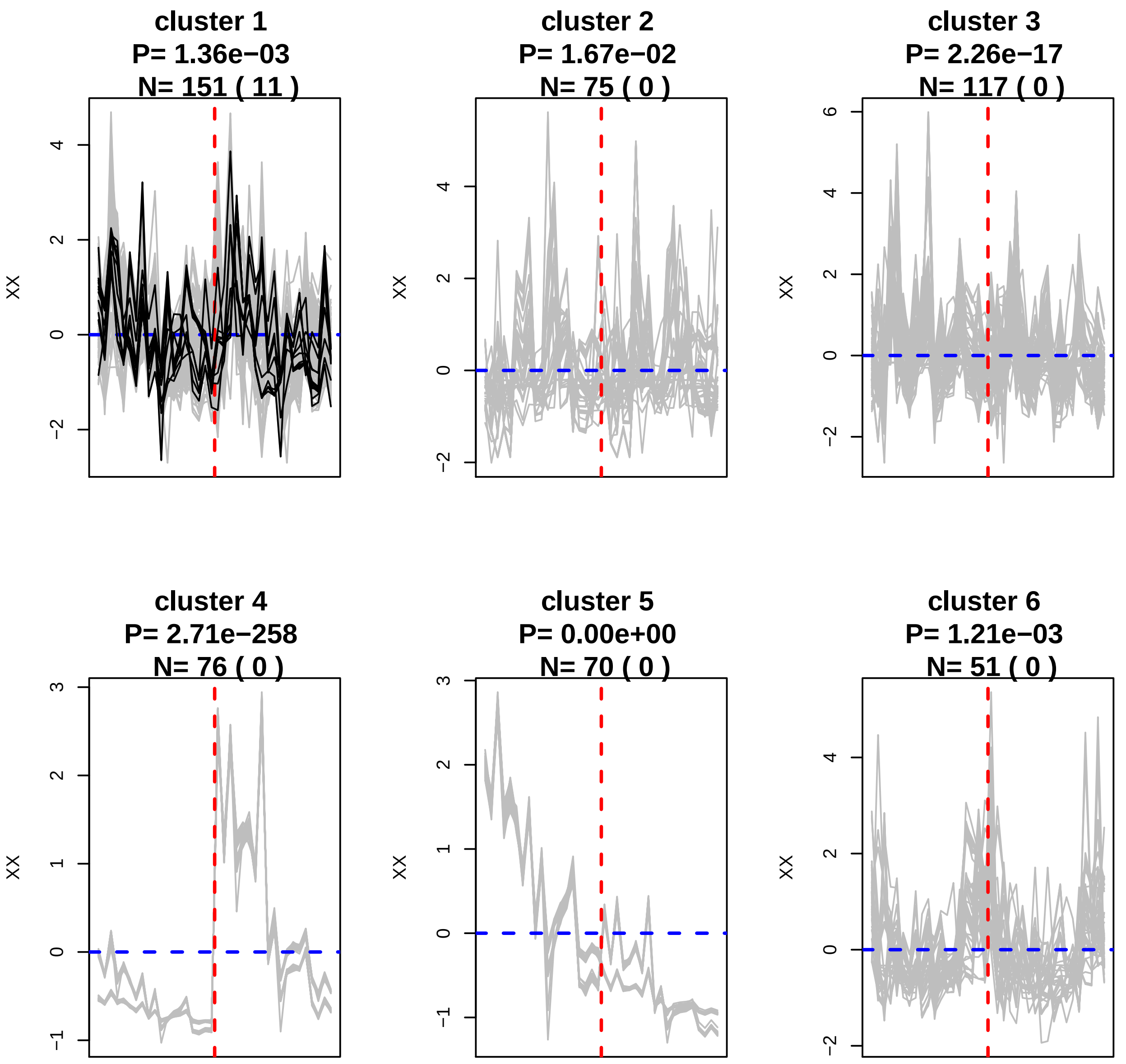

Experiment No. 8

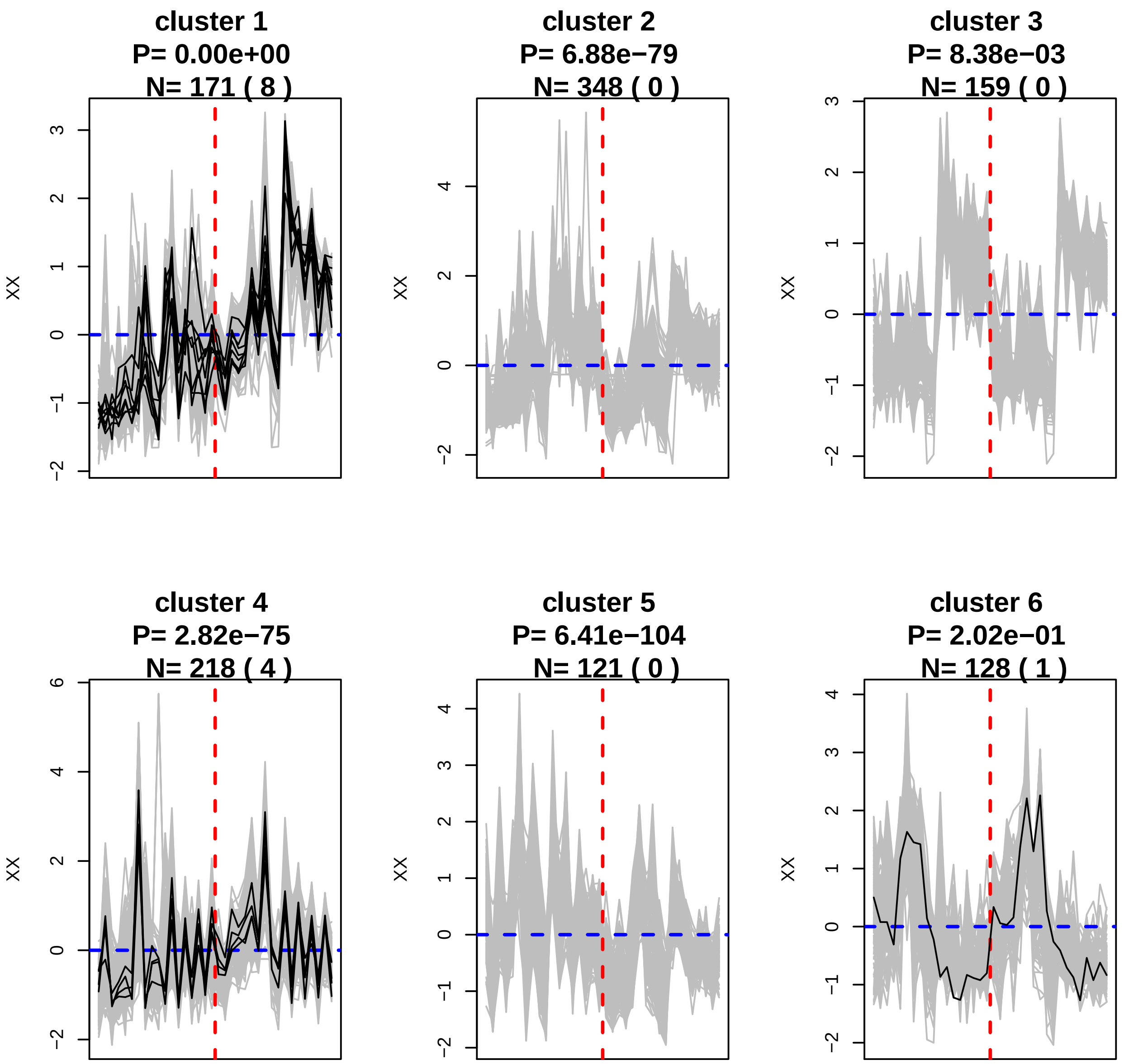

Experiment No. 9

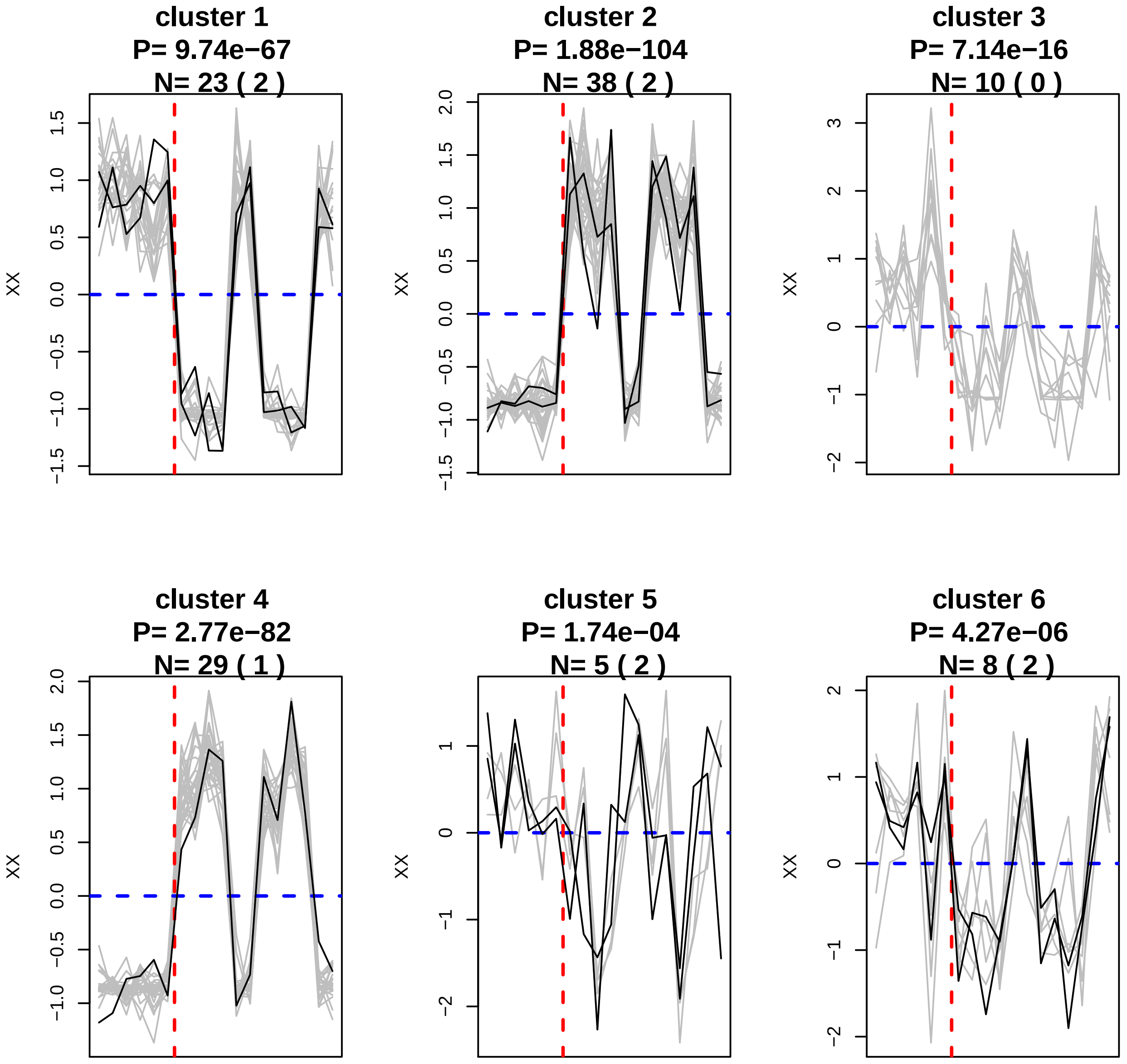

Experiment No. 10

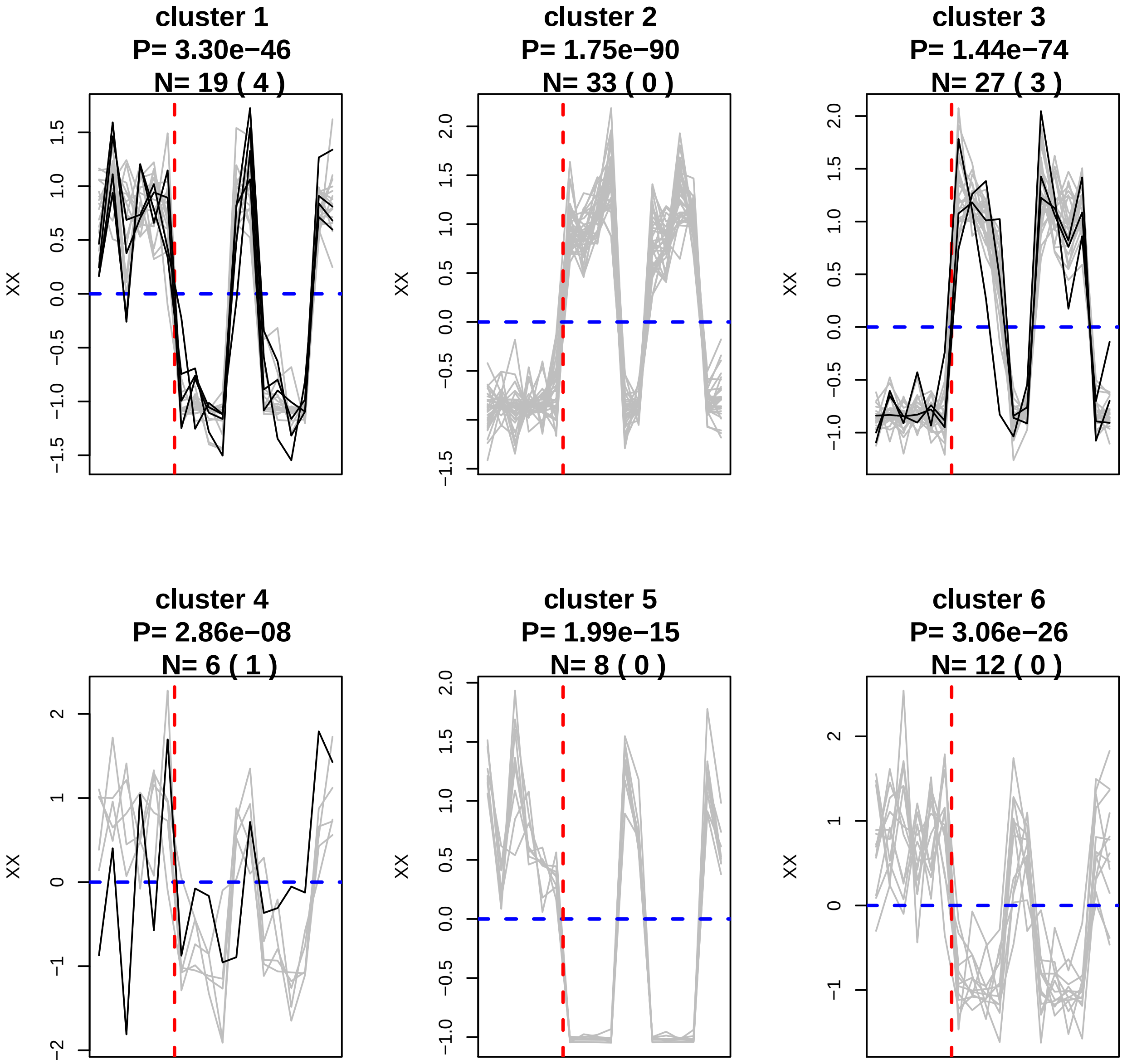

Experiment No. 11

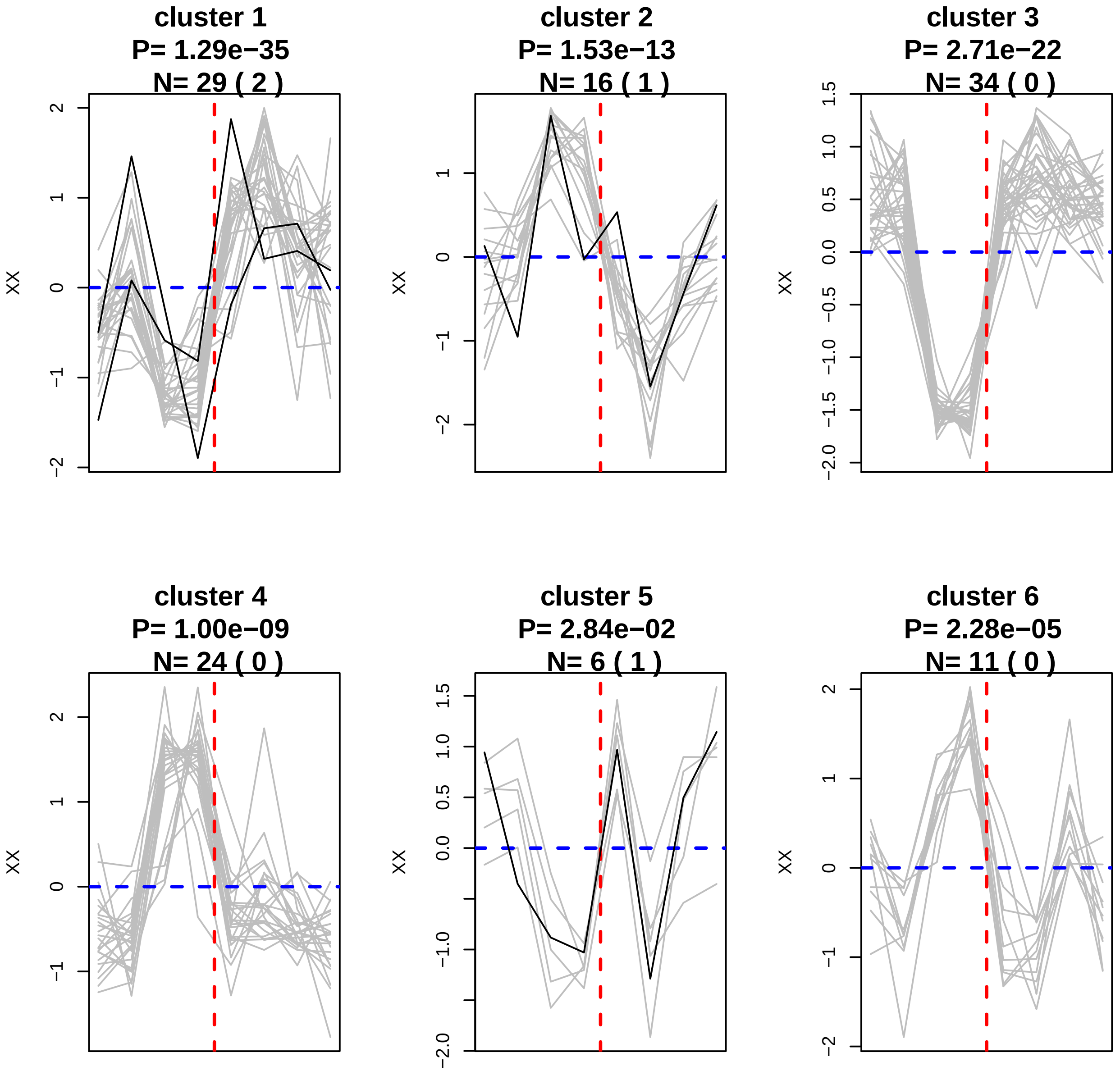

Figure S17: Expression profiles (grey) of genes selected by PCA or TD based unsupervised FE in 11 experiments. Genes are clustered in to six clusters with hierarchical clustering (Ward algorithm). Black lines are those of genes that are selected most frequently (more than 7 out of 11 experiments). Horizontal broken blue line represent base line and vertical broken red line represent boundary between control (left side) and treated (right side) samples. N is the number of genes within each cluster. The numbers in parentheses are those of most frequently selected genes. P-values are computed by t test where genes in each cluster are treated as a group. Profiles are standardized such that they have zero mean and unit variance.
