# Supplementary material for: Tensor Decomposition-Based Unsupervised Feature Extraction Can Identify the Universal Nature of Sequence-Nonspecific Off-Target Regulation of mRNA Mediated by MicroRNA Transfection
Source: Cells. 2018 Jun 4;7(6):54. doi: 10.3390/cells7060054 (PMC6025034; doi:10.3390/cells7060054)
Supplement: Supplementary file 1 [file cells-07-00054-s001.zip › Supp_Figures.pdf]

Supplementary figures cited in the text

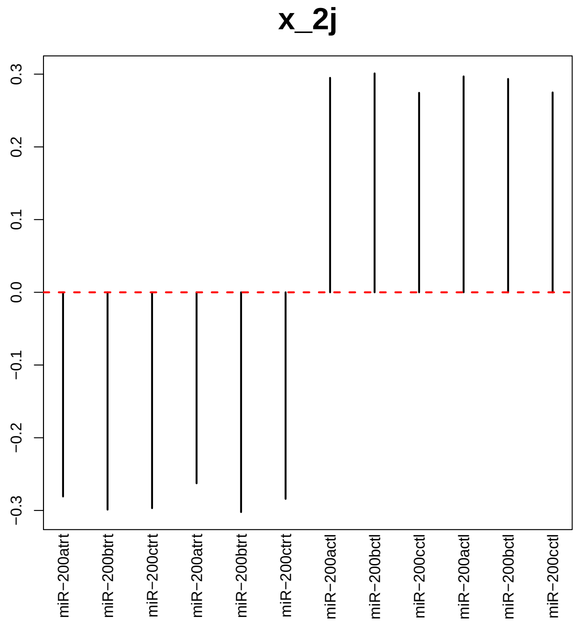

Figure S1: $x_{\ell_2=2,j}$ for experiment No.1

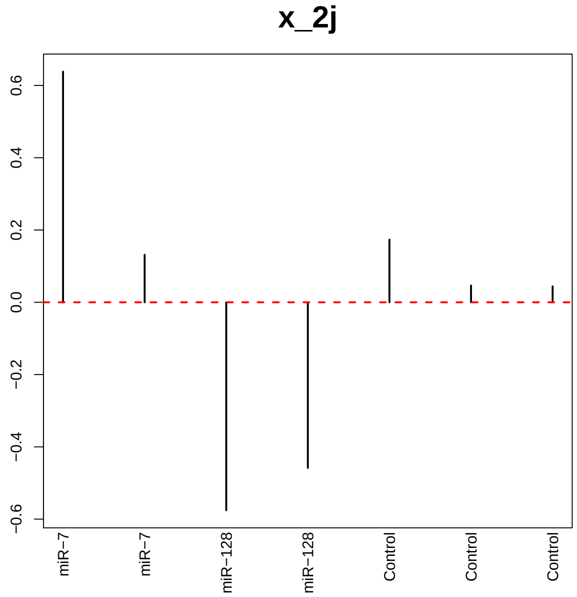

Figure S2: $x_{\ell_2=2,j}$ for experiment No.2

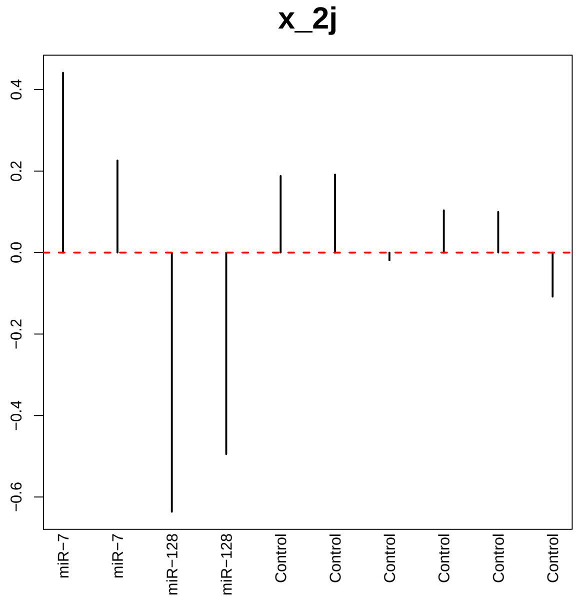

Figure S3: $x_{\ell_2=2,j}$ for experiment No.3

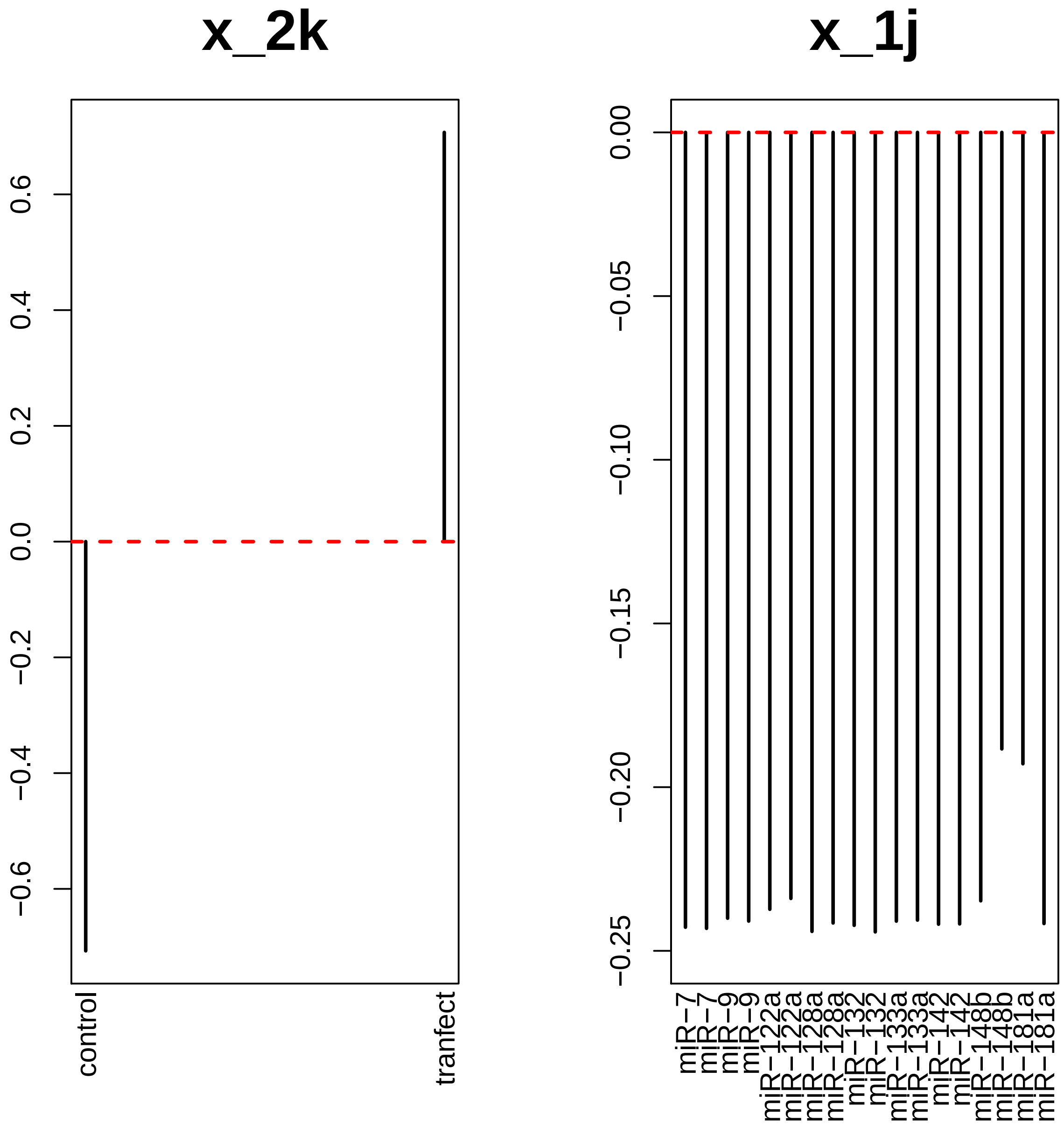

Figure S4: $x_{\ell_3=2,k}$ and $x_{\ell_2=1,j}$ for experiment No.4

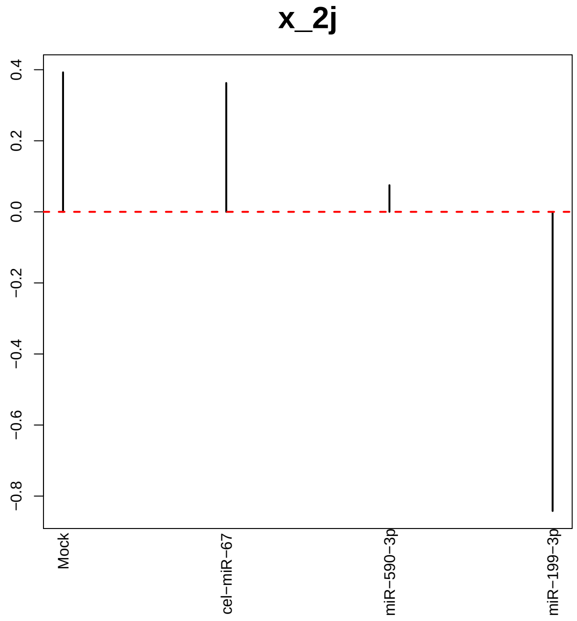

Figure S5: $x_{\ell_2=2,j}$ for experiment No.5

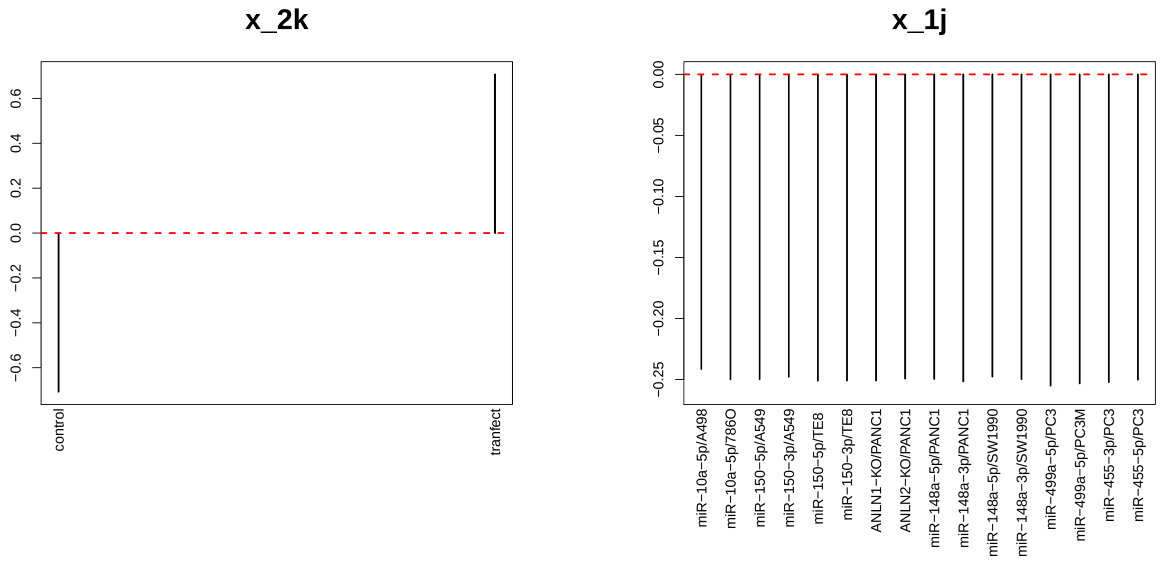

Figure S6: $x_{\ell_3=2,k}$ and $x_{\ell_2=1,j}$ for experiment No.6

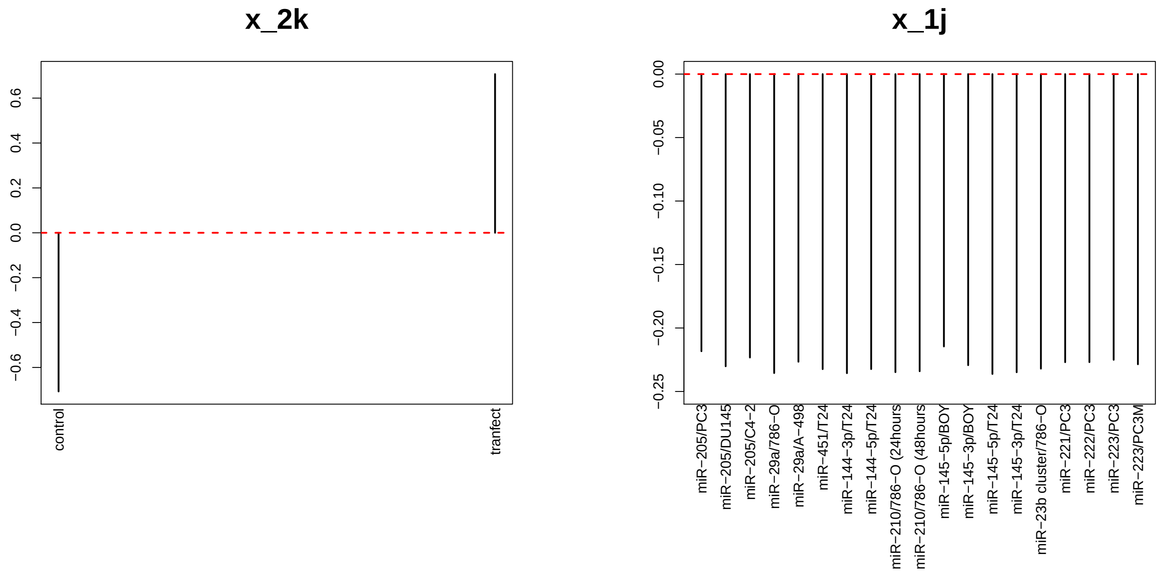

Figure S7: $x_{\ell_3=2,k}$ and $x_{\ell_2=1,j}$ for experiment No.7

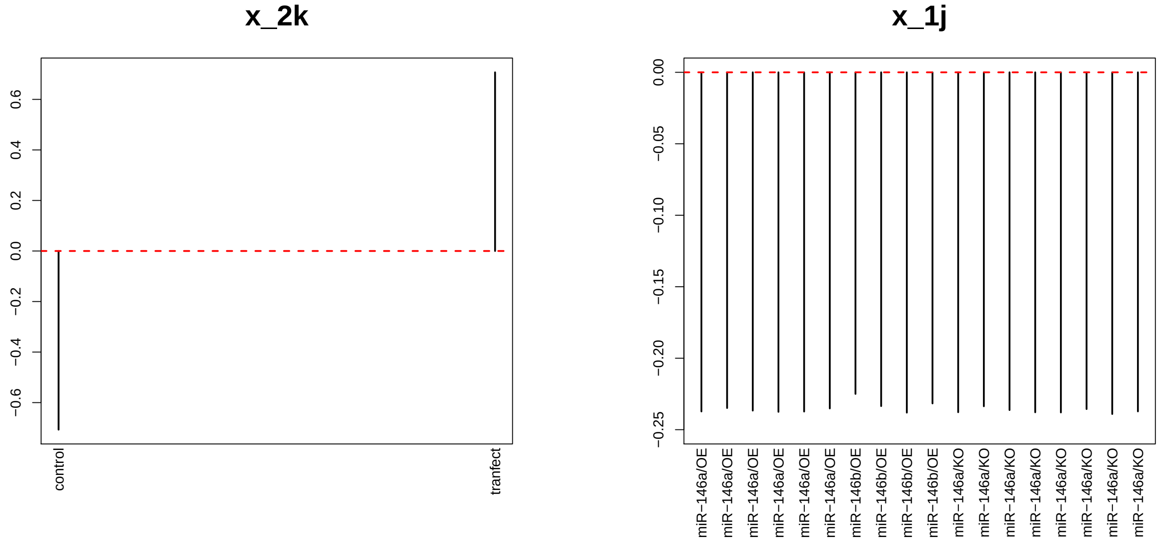

Figure S8: $x_{\ell_3=2,k}$ and $x_{\ell_2=1,j}$ for experiment No.8

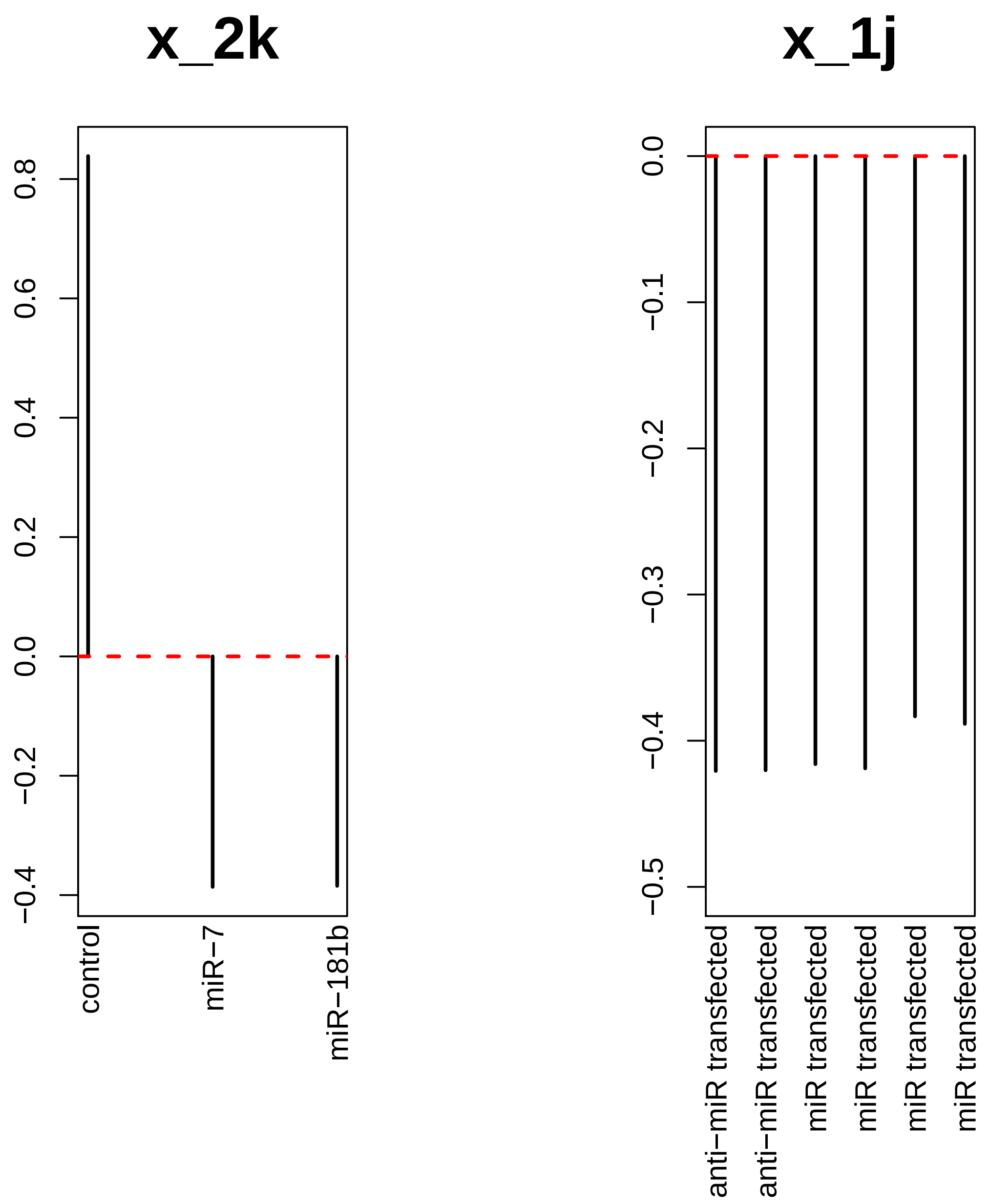

Figure S9: $x_{\ell_3=2,k}$ and $x_{\ell_2=1,j}$ for experiment No.9

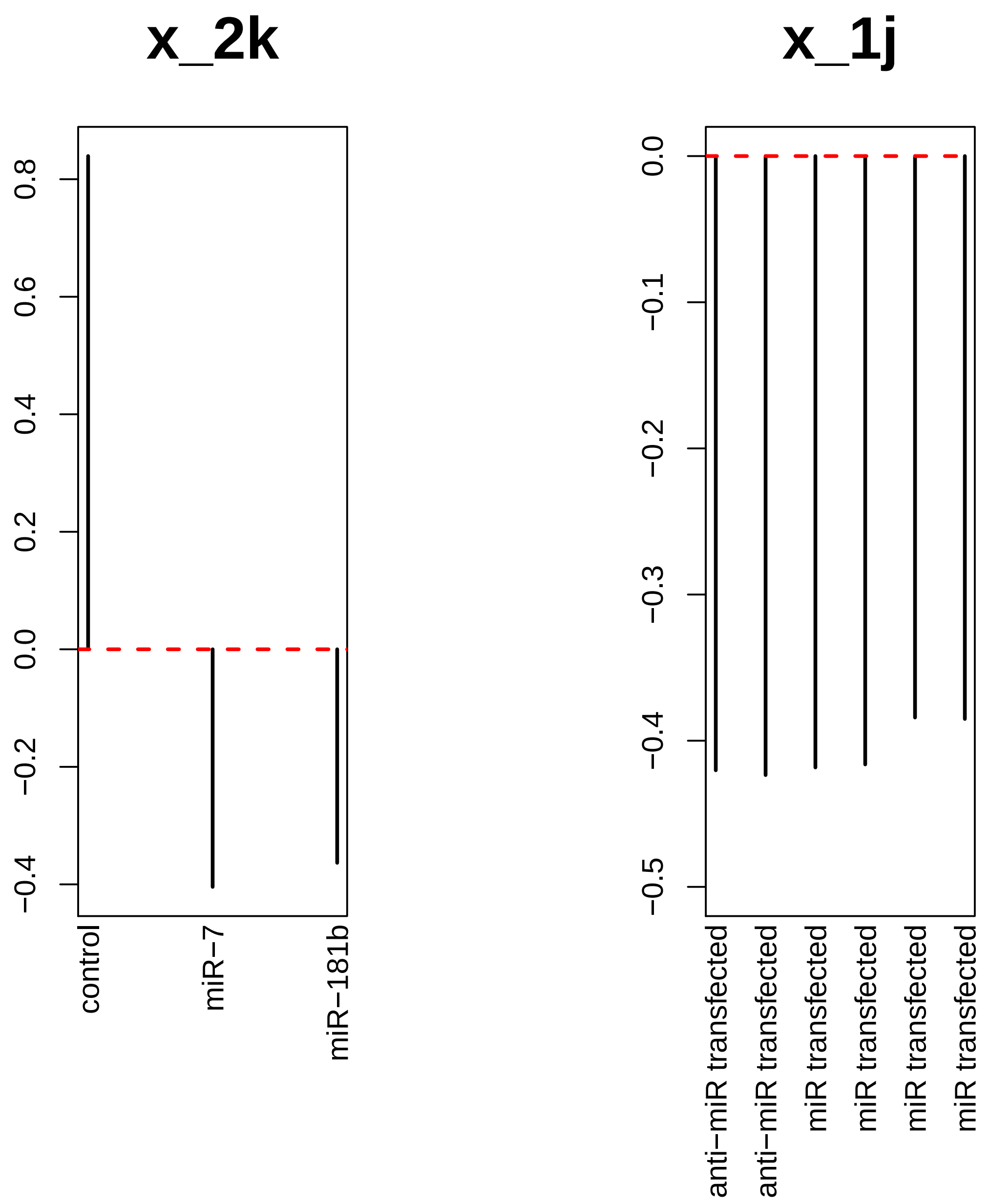

Figure S10: $x_{\ell_3=2,k}$ and $x_{\ell_2=1,j}$ for experiment No.10

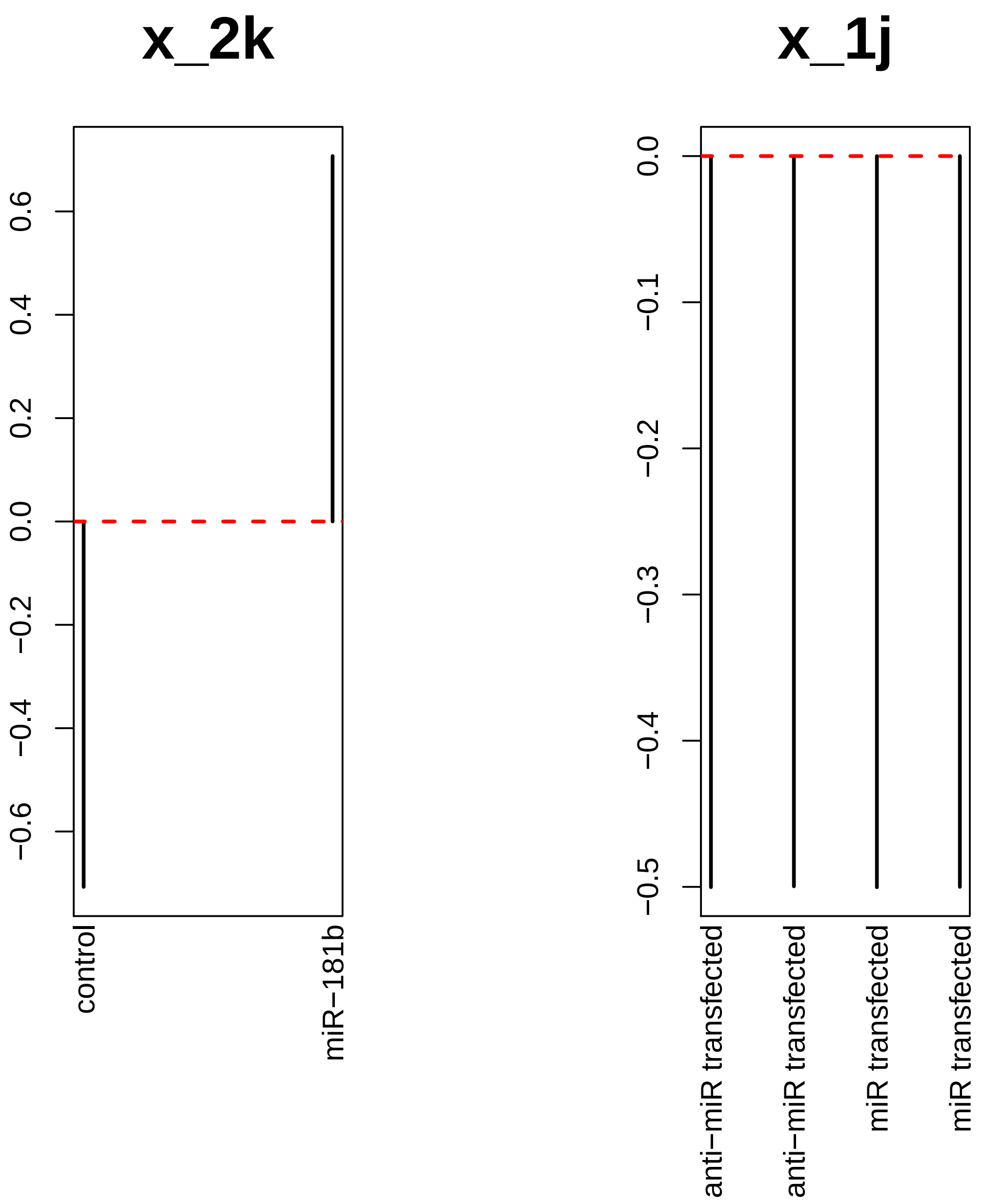

Figure S11: $x_{\ell_3=2,k}$ and $x_{\ell_2=1,j}$ for experiment No.11
